# Clonal expansion and epigenetic inheritance of long-lasting NK cell memory

**DOI:** 10.1038/s41590-022-01327-7

**Published:** 2022-10-26

**Authors:** Timo Rückert, Caleb A. Lareau, Mir-Farzin Mashreghi, Leif S. Ludwig, Chiara Romagnani

**Affiliations:** 1 Innate Immunity, Deutsches Rheuma-Forschungszentrum Berlin (DRFZ), ein Leibniz Institut, Berlin, Germany; 2 Department of Pathology, Stanford University, Stanford, CA, USA; 3 Therapeutic Gene Regulation, Deutsches Rheuma-Forschungszentrum Berlin (DRFZ), ein Leibniz Institut, Berlin, Germany; 4 Berlin Institute of Health at Charité Universitätsmedizin Berlin, Berlin, Germany; 5 Max-Delbrück-Center for Molecular Medicine in the Helmholtz Association (MDC), Institute for Medical Systems Biology (BIMSB), Berlin, Germany; 6 Charité—Universitätsmedizin Berlin, Corporate Member of Freie Universität Berlin and Humboldt Universität zu Berlin, Medizinische Klinik für Gastroenterologie, Infektiologie und Rheumatologie, Berlin, Germany; 7 Leibniz-Science Campus Chronic Inflammation, Berlin, Germany

## Abstract

Clonal expansion of cells with somatically diversified receptors and their long-term maintenance as memory cells is a hallmark of adaptive immunity. Here, we studied pathogen-specific adaptation within the innate immune system, tracking natural killer (NK) cell memory to human cytomegalovirus (HCMV) infection. Leveraging single-cell multiomic maps of ex vivo NK cells and somatic mitochondrial DNA mutations as endogenous barcodes, we reveal substantial clonal expansion of adaptive NK cells in HCMV^+^ individuals. NK cell clonotypes were characterized by a convergent inflammatory memory signature enriched for AP1 motifs superimposed on a private set of clone-specific accessible chromatin regions. NK cell clones were stably maintained in specific epigenetic states over time, revealing that clonal inheritance of chromatin accessibility shapes the epigenetic memory repertoire. Together, we identify clonal expansion and persistence within the human innate immune system, suggesting that these mechanisms have evolved independent of antigen-receptor diversification.

Vertebrates have evolved several mechanisms of immune adaptation, enabling optimized secondary responses to the same challenge. In a traditional paradigm, persistent immune memory has exclusively been associated with T and B cell clones selected from a pool expressing enormously diversified antigen receptors. Competitive clonal expansion after antigen encounter is accompanied by pronounced epigenetic remodeling, forming a repertoire of memory cells with enhanced and accelerated recall responses^1^. The dogma that adaptation mechanisms are exclusively confined to B and T cells has been challenged by observations of adjusted secondary responses within the innate immune system, with the most prominent example being ‘trained immunity’ of myeloid cells^2^. As for adaptive immune cells, these functional differences are epigenetically imprinted but are generally considered to be more transient and shorter lived than classical adaptive immunity. However, long-term maintenance of trained immunity has been observed in different cell types^3–6^. Further, in sharp contrast with the massive expansion of individual, epitope-specific T and B cell clones, trained immunity is mostly described at the population level, as innate immune cell activation occurs via widely expressed pattern recognition receptors.

More specific innate recognition mechanisms for several pathogens are used by natural killer (NK) cells, with the most prominent example being cytomegalovirus (CMV)^7^. CMV closely interacts with the host immune system to establish lifelong latent infection^8^. Consequently, CMV infection strongly imprints both the innate and adaptive immune repertoires, resulting in expansion of T cell clones and the emergence of ‘adaptive’ NK cells. While in C57BL/6 mice, these cells are expanded in response to the engagement of the activating receptor Ly49H by the murine CMV ligand m157 (ref. ^9^), human adaptive NK cells specifically recognize peptides derived from the human CMV (HCMV) protein gpUL40 through the receptor CD94/NKG2C, facilitating affinity-dependent expansion of NKG2C^+^ NK cells^10^. Recognition via conserved innate receptors expressed by cell populations is an important difference to unique antigen receptors, likely affecting the size of the pool recruited into the response. In this context, the narrow, self-major histocompatibility complex (MHC)-specific killer cell immunoglobulin-like receptor (KIR) profile of human adaptive NK cells has sparked discussions on an oligoclonal origin^11^. Along this line, animal transfer studies of barcoded NK cells or progenitors have demonstrated the potential of NK cells to clonally expand in immunodeficient or depleted hosts^12–14^, raising conceptual parallels to adaptive immune cells^15^. However, how these findings apply to natural infection in healthy individuals, where much larger populations of cells compete with each other and clonal diversity is not biased by transfer efficiency, is still unclear. Moreover, whether adaptive NK cell expansions induced by HCMV emerge from oligoclonal ‘founder’ populations and persist to form long-lasting memory remains elusive.

Here, we generated a single-cell multiomic map of human NK cells from HCMV^+^ and HCMV^−^ individuals, integrating measurements of gene expression and chromatin accessibility with lineage tracing using somatic mitochondrial DNA (mtDNA) mutations as endogenous barcodes^16,17^. We detected substantial clonal expansion of adaptive NK cells with a convergent inflammatory memory signature accompanied by private, clonotype-associated accessible chromatin sites, demonstrating a human innate immune population clonally persisting in a memory state.

## Results

### Mapping NK cell subsets onto transcriptional and epigenetic landscapes

**Extended Data Fig. 1:**
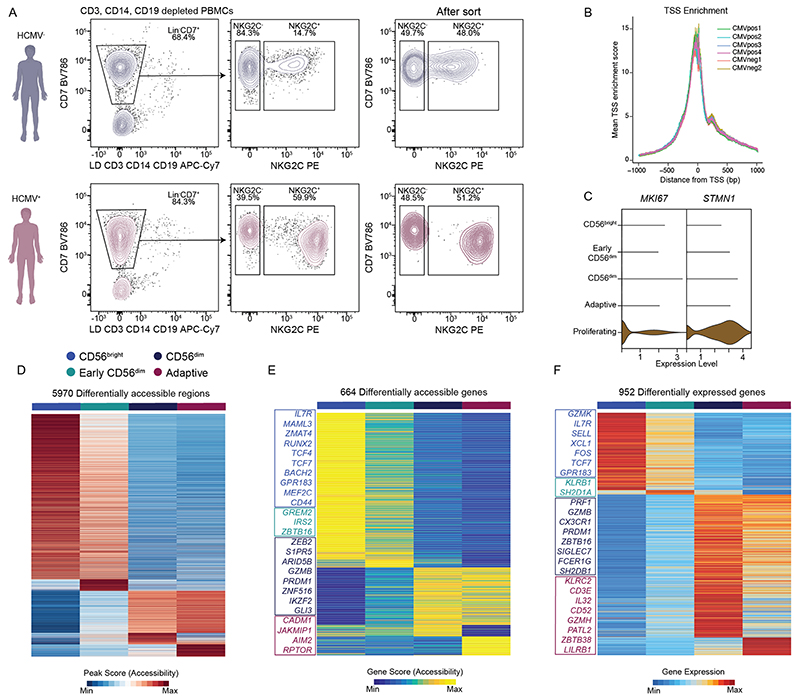
Mapping NK cell subsets onto transcriptional and epigenetic landscapes. **(a)** Sorting strategy for one representative HCMV^−^ and HCMV^+^ donor, respectively. **(b)** Transcription start site (TSS) enrichment plot overlaid for all donors. **(c)** Expression of proliferation genes *MKI67* and *STMN1*. **(d-f)** Row-scaled differentially accessible regions (D), genes (E) and differentially expressed genes (F) per cluster. Exemplary cluster-associated genes are listed in the respective colors. **(h)** Gene accessibility, imputed expression (violins), and their Pearson correlation (indicated as links) for *RUNX2* and *ZEB2*. Created with BioRender.com.

**Extended Data Fig. 2:**
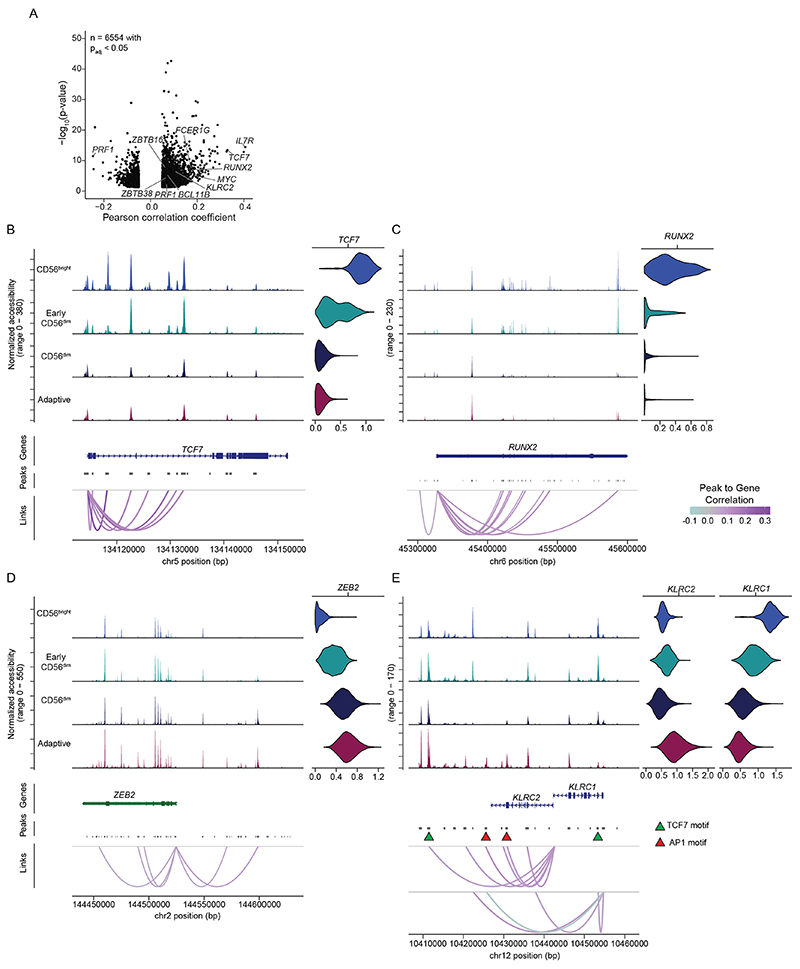
Integration of chromatin accessibility and gene expression. **(a)** Pearson correlation between open chromatin regions and gene expression, p-value from one-sided z-test of background-normalized correlation-coefficient as implemented in Signac^62^.**(b-e)** Chromatin accessibility, imputed expression (violins) and their Pearson correlation (indicated as links) for indicated genes *TCF7* (B), *RUNX2* (C), *ZEB2* (D), *KLRC2* and *KLRC1* (E).

**Fig. 1:**
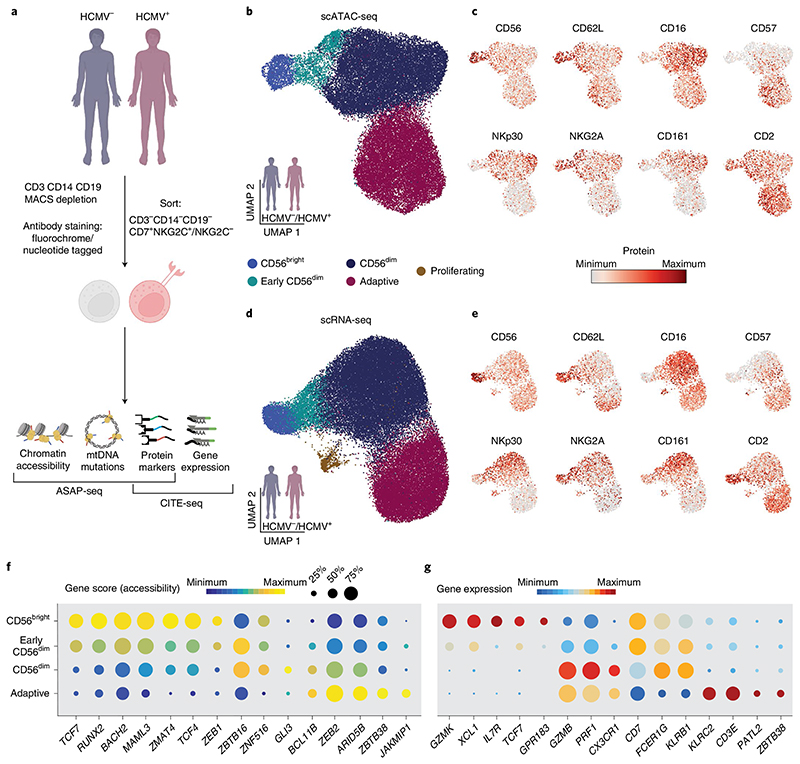
Mapping NK cell subsets onto transcriptional and epigenetic landscapes. **a**, NKG2C^+^ and NKG2C^−^ NK cells were isolated from four to five HCMV^+^ individuals and two HCMV^−^ healthy blood donors, stained with nucleotide barcode-labeled antibodies, mixed at a 1:1 ratio and analyzed by ASAP-seq *(n*= 6) and CITE-seq *(n* = 7). **b**,**d**, Integrated UMAP embedding of NK cells from donors analyzed by scATAC-seq (**b**; *n* = 6) and scRNA-seq (**d**; *n* = 7). **c**,**e**, Surface protein expression of the indicated markers as measured by ASAP-seq (**c**) or CITE-seq (**e**). **f**,**g**, Column-scaled accessibility scores (**f**) and expression (**g**) per cluster and detection frequencies for the indicated genes. Created with BioRender.com.

To resolve the relationship of human NK cell subsets and the impact of HCMV on these populations, we performed single-cell assay of transposase-accessible chromatin (ATAC) with select antigen profiling by sequencing (ASAP-seq)^18^ and cellular indexing of transcriptomes and epitopes (CITE-seq)^19^ of NK cells from peripheral blood of HCMV^+^ donors with NKG2C^+^ NK cell expansions and HCMV^−^ individuals (Fig. 1a and Supplementary Table 1). NKG2C^+^/NKG2C^−^ NK cells (sorted as CD3^−^CD14^−^CD19^−^CD7^+^; Methods) from all donors were equally enriched to a 1:1 ratio to correct for different frequencies of NKG2C^+^ cells between HCMV^+^ and HCMV^−^ individuals (Extended Data Fig. 1a). After filtering for high-quality cells (Methods), 39,106 cells and 49,530 cells were analyzed for their epigenetic and transcriptional profiles, respectively. We detected expression of 19,975 genes and accessibility of 147,299 chromatin regions with consistent enrichment of transcription start sites (TSSs) across donors (Extended Data Fig. 1b). Using cell surface protein information, we annotated clusters defined by their transcriptomes and chromatin accessibility with long-standing NK cell subset definitions, while at the same time testing the validity of these population definitions in an unbiased manner (Fig. 1b–e). Namely, we observed four main clusters in both modalities that could be designated as CD56^bright^, early CD56^dim^, mature CD56^dim^ and adaptive NK cells (Fig. 1b,d) with clearly distinct genomic signatures (Extended Data Fig. 1d–f), in line with previous data^20–24^. An additional cluster of proliferating cells was only resolved via transcriptional profiling (Fig. 1d and Extended Data Fig. 1c). CD56^bright^ (CD16^−/lo^CD57^−^CD62L^+^NKG2A^+^) cells were characterized by high accessibility around loci of characteristic transcription factors (TFs) *TCF7, RUNX2, BACH2, ZEB1* and *MAML3*, and expression of signature transcripts, including *GZMK, XCL1* and *IL7R*. By contrast, CD56^dim^ (CD16^+^CD57^+^/CD57^−^CD62L^+^/CD62L^−^NKG2A^+^/NKG2A^−^) NK cells were characterized by chromatin accessibility in the proximity of *ZBTB16, BCL11B, GLI3* and *ZEB2* and expression of *GZMB, PRF1* and *CX3CR1*. Between CD56^bright^ and CD56^dim^ NK cells, a population of seemingly intermediate, early CD56^dim^ (CD16^+^CD57^−^CD62L^+^NKG2A^+^) NK cells shared signatures of both subsets, such as high gene scores for *TCF7, ZEB1* and *ZBTB16* (Fig. 1b–g and Extended Data Fig. 1d–f). Label transfer between modalities further allowed for directly linking gene expression and chromatin accessibility, generating an integrated resource that enables correlation-based prediction of subset-specific gene regulatory elements for core regulators of NK cell identity, such as *TCF7, RUNX2* and *ZEB2* (Extended Data Fig. 2a–d). Overall, we provide a multimodal map of human NK cells, combining single-cell measurements of chromatin accessibility, gene expression and surface phenotypes.

### Distinct signatures for naive and adaptive NKG2C^+^ NK cells

Our multiomic analysis of human NK cells clearly revealed the presence of a separate and genomically distinct adaptive NK cell cluster (Fig. 1b–g), characterized by high transcript levels of *KLRC2, CD3E* and *PATL2* together with marked downregulation of *CD7, KLRB1* and *FCER1G* (Fig. 1g) and distinct surface marker expression of CD57 and CD2 coinciding with low NKp30, CD161 and NKG2A (Fig. 1c,e). While adaptive NK cells shared large parts of their genomic effector signature with conventional CD56^dim^ NK cells, they displayed a marked reduction of *ZBTB16* and *ZNF516* and further increased gene scores for *ZEB2* and the metabolic regulator *ARID5B^25^* compared to CD56^dim^ NK cells (Fig. 1f). Moreover, adaptive NK cells were characterized by expression and accessibility of *ZBTB38* and *JAKMIP1* (Fig. 1f,g and Extended Data Fig. 1e,f). Importantly, the integrated analysis demonstrated remodeling at chromatin regions around the *KLRCX* locus, with several of those being positively correlated with increased *KLRC2* expression and one region having a negative correlation with *KLRC1* expression (Extended Data Fig. 2e). Their functional role was supported by the presence of several TF binding motifs, with the most strongly correlating putative *KLRC1* enhancer containing a TCF7 motif, consistent with expression and accessibility in CD56^bright^ NK cells, while AP1 motifs were notable in the putative *KLRC2* enhancers specifically accessible in adaptive NK cells, which together may regulate the core adaptive NK cell phenotype characterized by high surface expression of NKG2C combined with lack of NKG2A^26^.

**Fig. 2:**
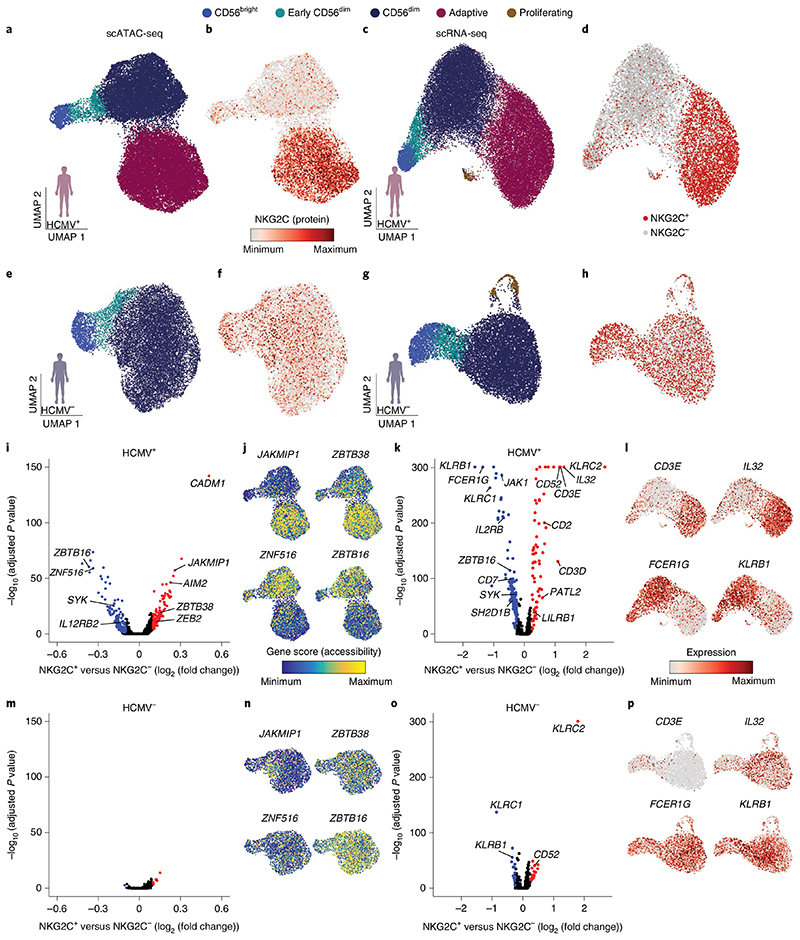
Distinct signatures of naive and adaptive NKG2C^+^ NK cells. **a**,**e**, UMAP embedding of NK cells from HCMV^+^ (**a**; *n = 4)* and HCMV^−^ (**e**; *n* = 4) donors analyzed by scATAC-seq. **b**,**f**, NKG2C surface expression as measured by ASAP-seq. **c**,**g**, UMAP embedding of NK cells from HCMV^+^ (**c**; *n* = 5) and HCMV^−^(**g**; *n* = 2) donors analyzed by scRNA-seq. **d**,**h**, Distribution of barcoded NKG2C^+^ and NKG2C^−^ populations. **i**,**m**, Differentially accessible genes between NKG2C^+^ and NKG2C^−^ NK cells for HCMV^+^ (**i**) and HCMV^−^ (**m**) donors as determined by logistic regression. **j**,**n**, Representative accessibility scores of genes defining adaptive NK cells for HCMV^+^ (**j**) and HCMV^−^ (**n**) donors. **k**,**o**, Differentially expressed genes between NKG2C^+^ and NKG2C^−^ NK cells for HCMV^+^ (**k**) and HCMV^−^ (**o**) donors determined by two-sided Wilcoxon rank-sum test with Bonferroni adjustment. **l**,**p**, Representative expression of genes defining adaptive NK cells for HCMV^+^ (**l**) and HCMV^−^ (**p**) donors. Created with BioRender.com.

While expansion of adaptive NKG2C^+^ NK cells is associated with HCMV infection, NKG2C^+^ NK cells are present also in HCMV^−^ individuals, albeit at lower frequencies. To directly compare the transcriptional and epigenetic landscape of NKG2C^+^/NKG2C^−^ NK cells and their distribution within NK cell clusters in HCMV^+^ and HCMV^−^ individuals, we divided the datasets by HCMV serostatus (Fig. 2a–h). As expected, the adaptive cluster was selectively present in HCMV^+^ individuals and was mostly comprised of NKG2C^+^ NK cells (Fig. 2a–d). Comparative analysis of chromatin accessibility and gene expression after downsampling to equal cell numbers for both donor groups highlighted the specific impact of HCMV on NKG2C^+^ cells. A total of 232 genes were differentially accessible between the NKG2C^+^ and NKG2C^−^ populations in HCMV^+^ individuals, including key adaptive genes, such as *JAKMIP1, ZBTB38, ZBTB16* or *ZNF516* (Fig. 2i), along with 269 differentially expressed genes, including adaptive NK cell markers *CD3E, IL32, FCER1G* and *KLRB1* (Fig. 2k). Conversely, NKG2C^+^ NK cells from HCMV^−^ donors were randomly dispersed among NKG2C^−^ NK cells (Fig. 2e–h), and differences in chromatin accessibility and gene expression, apart from *KLRC2* (encoding NKG2C), were minor (Fig. 2m,o), highlighting their largely similar epigenomes and transcriptomes. Genes differentially accessible or expressed between NKG2C^+^ and NKG2C^−^ cells in HCMV^+^ individuals, including *ZBTB16* and *FCER1G*, followed a coordinated pattern separating conventional from adaptive NK cells, whereas this was not observed in HCMV^−^ individuals (Fig. 2j,n,l,p), underlining that these differences reflect the adaptive remodeling induced in NKG2C^+^ cells by HCMV.

**Extended Data Fig. 3:**
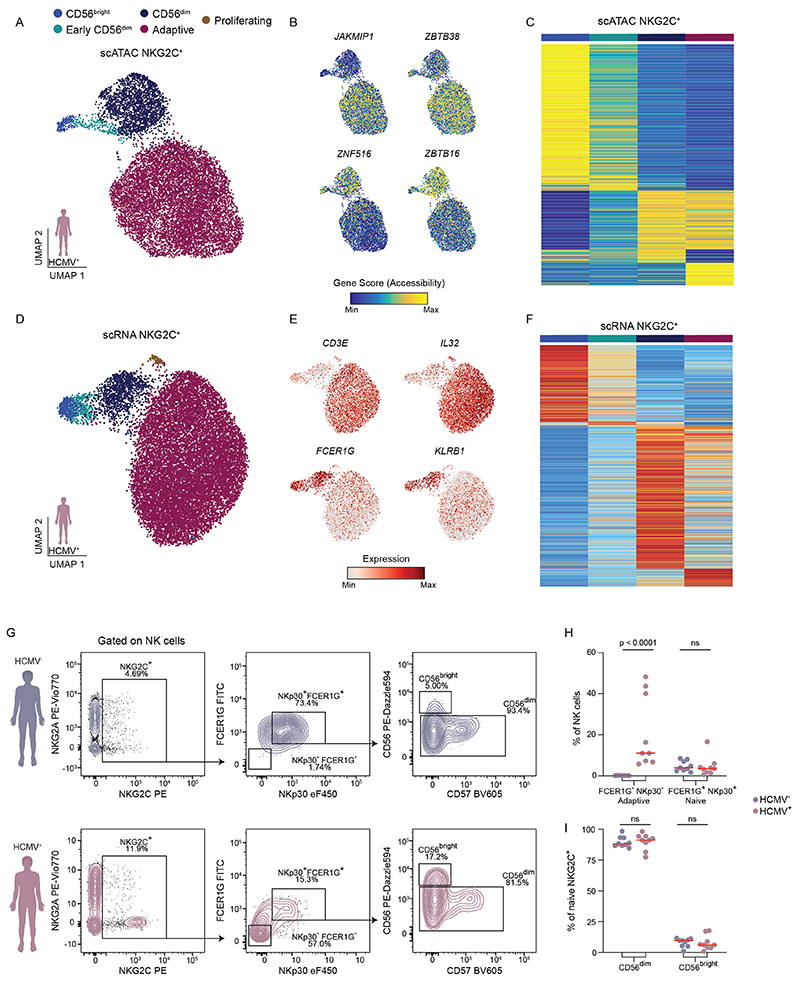
Distinct signatures dissect NKG2C*NK cells into naïve and adaptive. (**a** + **d**) UMAP embedding of scATAC-seq (A) and scRNA-seq (D) of NKG2C^+^ NK cells from HCMV^+^ individuals. **(b** + **e)** Accessibility (B) and expression (E) of key genes regulated in adaptive NK cells. **(c** + **f)** Row-scaled accessibility (C) and expression (F) of NK cell subset-specific signatures from fully integrated datasets (Fig. 1) in NKG2C^+^ NK cell clusters from HCMV^+^ donors. **(g−i)** Representative expression (G) and frequencies (H) of FCER1G^+^ NKp30^+^(‘Naive’) and FCER1G^-^ NKp30^−^ (‘Adaptive’) NKG2C^+^ NK cells and distribution of naïve NKG2C^+^ NK cells into CD56^bright^ and CD56^dim^ subsets (I) in HCMV^−^ (n = 9) and HCMV^+^ (n = 9) donors. Kruskal-Wallis test with Dunn’s post-hoc test. Created with BioRender.com.

Interestingly, even HCMV^+^ individuals displayed a small fraction of NKG2C^+^ NK cells that were present in all three subsets of the conventional NK cell compartment (Fig. 2a–d). Indeed, separate analysis of NKG2C^+^ NK cells alone from HCMV^+^ individuals revealed the coexistence of adaptive NKG2C^+^ NK cells with a minority of ‘naive’ NKG2C^+^NK cells (Extended Data Fig. 3a,d) that lacked adaptive remodeling (Extended Data Fig. 3b,e) and spanned the whole spectrum of conventional NK cells, recapitulating the signatures identified from the full integrated analysis (Extended Data Fig. 3c,f). We confirmed by flow cytometry that the frequencies of naive NKG2C^+^ NK cells were comparable between HCMV^+^ and HCMV^−^ individuals and that they were similarly distributed between the CD56^bright^ and CD56^dim^ compartment (Extended Data Fig. 3g–i).

Together, this comparative analysis of HCMV^+^ and HCMV^−^ donors highlighted the pronounced remodeling that HCMV imposes on the NKG2C^+^ NK cell pool that coexists with a minority of naive NKG2C^+^ NK cells during the latent phase of HCMV infection.

### HCMV leaves an inflammatory memory footprint enriched in AP1 motifs

**Extended Data Fig. 4:**
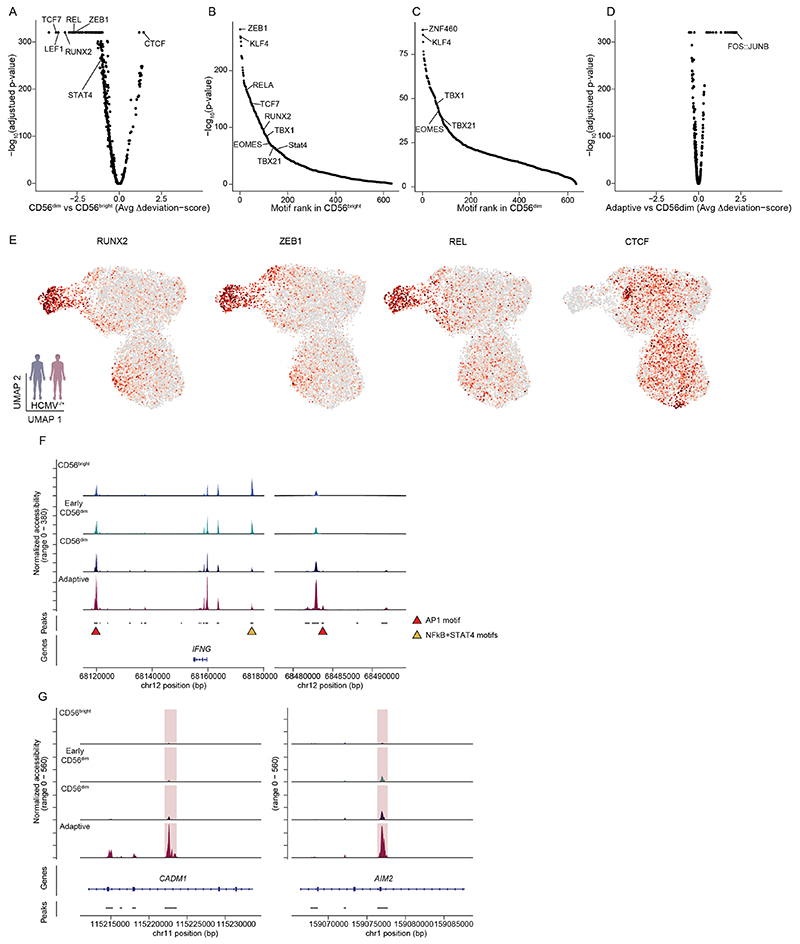
HCMV infection leaves a persistent chromatin footprint enriched for AP1 motifs. **(a)** Differentially active motifs between CD56^dim^ and CD56^bright^ NK cells by Wilcoxon rank sum test. **(b-c)** Motif enrichment in chromatin regions specifically accessible in CD56^bright^ (B) and CD56^dim^ NK cells as determined by hypergeometric test. **(d)** Differentially active motifs between adaptive and CD56^dim^ NK cells by Wilcoxon rank sum test. **(e)** Activity of the respective TF motifs projected onto UMAP embedding. **(f)** Accessibility of *IFNG*-associated open-chromatin regions containing AP1 or STAT4 and NFκB motifs. **(g)** Per cluster accessibility of AP1-motif containing chromatin regions in the proximity of *CADM1* and *AIM2*. Created with BioRender.com.

**Fig. 3:**
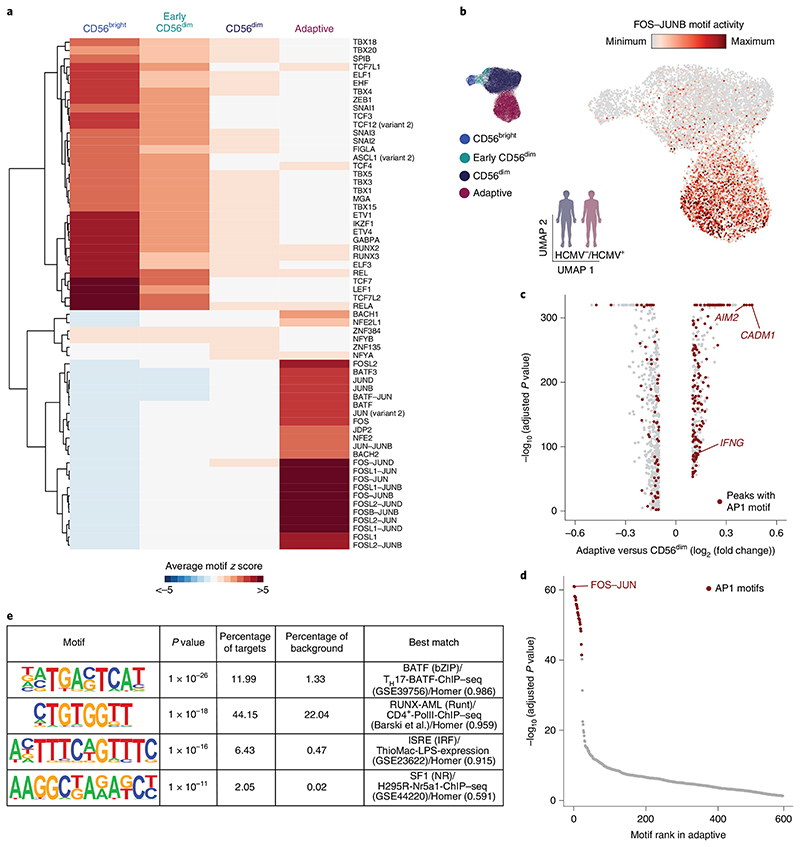
HCMV infection leaves an inflammatory memory footprint enriched in AP1 motifs. **a**, Heat map of differentially active motifs represented as average chromVAR deviation scores per cluster. **b**, FOS–JUNB motif activity projected on UMAP embedding. **c**, DARs between adaptive and CD56^dim^ NK cells determined by two-sided Wilcoxon rank-sum test with Bonferroni adjustment. AP1-motif-containing regions are marked in red, and selected regions are annotated by gene proximity. **d**, Motif enrichment in chromatin regions specifically accessible in adaptive NK cells as determined by one-sided hypergeometric test with Benjamini–Hochberg adjustment. **e**, De novo motif analysis results on chromatin regions specifically accessible in adaptive NK cells. *P* values were calculated by comparing enrichment to the cumulative binomial distribution. Created with BioRender.com.

While gene expression profiles and accessibility of regulatory elements in the proximity of *trans*-acting factors provide a view on the current state of a cell, analysis of TF motif enrichment in *cis*-regulatory elements also captures molecular events that have left footprints in the generation of this state. Global analysis using chromVAR^27^ identified a strong signature in CD56^bright^ NK cells with enhanced activity of motifs for TFs such as TCF7, RUNX2 or ZEB1 (Fig. 3a and Extended Data Fig. 4a,b,e), consistent with an important role of these TFs in shaping the epigenetic and transcriptional identity^21,24^ of this subset (Fig. 1f,g) and increased motif activity for NF-κB-related TFs (REL) and STAT4 (Fig. 3a and Extended Data Fig. 4a,b,e), reflecting the enhanced responsiveness of CD56^bright^ NK cells to activation by proinflammatory cytokines^28^. Accordingly, an NF-κB- and STAT4-motif-containing peak in the proximity of the *IFNG* gene was specifically accessible in CD56^bright^ cells, while this was closed in the more mature subsets (Extended Data Fig. 4f). While CD56^dim^ NK cells were mostly characterized by a lack of the CD56^bright^-specific TF motif activity, both CD56^dim^ and adaptive NK cells were further distinguished from CD56^bright^ NK cells by enhanced CTCF motif activity (Extended Data Fig. 4a,e), as previously described^24^. Importantly, adaptive NK cells showed a strikingly separated signature compared to both conventional NK cell subsets, characterized by enhanced activity of AP1 motifs, with the strongest difference observed for FOS–JUNB (Fig. 3a,b and Extended Data Fig. 4d), and AP1 motifs were the most significantly enriched in adaptive NK-cell-specific accessible regions (Fig. 3c,d). De novo motif prediction on these peaks also yielded a canonical AP1 motif as the most significant hit, further corroborating these results (Fig. 3e). Recently, AP1 motifs have been described to be at the core of a unifying inflammatory memory signature in different immune cell types and murine epithelial stem cells, the latter characterized by specific accessibility in the proximity of the *Aim2* and *Cadm1* loci^4^. Notably, their human orthologs also showed highly specific opening of AP1-motif-containing regions in adaptive NK cells (Fig. 3c and Extended Data Fig. 4g) and were among the genes with the most pronounced difference in accessibility between NKG2C^+^ and NKG2C^−^ cells in HCMV^+^ individuals (Fig. 2i). Finally, among the more accessible peaks with AP1 motifs were several enhancers of the *IFNG* gene (Extended Data Fig. 4f), providing a possible mechanism for the increased interferon-γ (IFNγ) production by adaptive NK cells in response to activating receptor stimulation^29,30^. Overall, these findings demonstrate how differential TF activity shapes NK cell epigenetic remodeling during differentiation and identify AP1 TFs as potential drivers shaping adaptive NK cell chromatin accessibility, an intriguing parallel to the inflammatory imprinting used to establish immune memory in different cell types^4,31,32^.

### Synergistic imprinting by HCMV peptides and proinflammatory cytokines

**Fig. 4:**
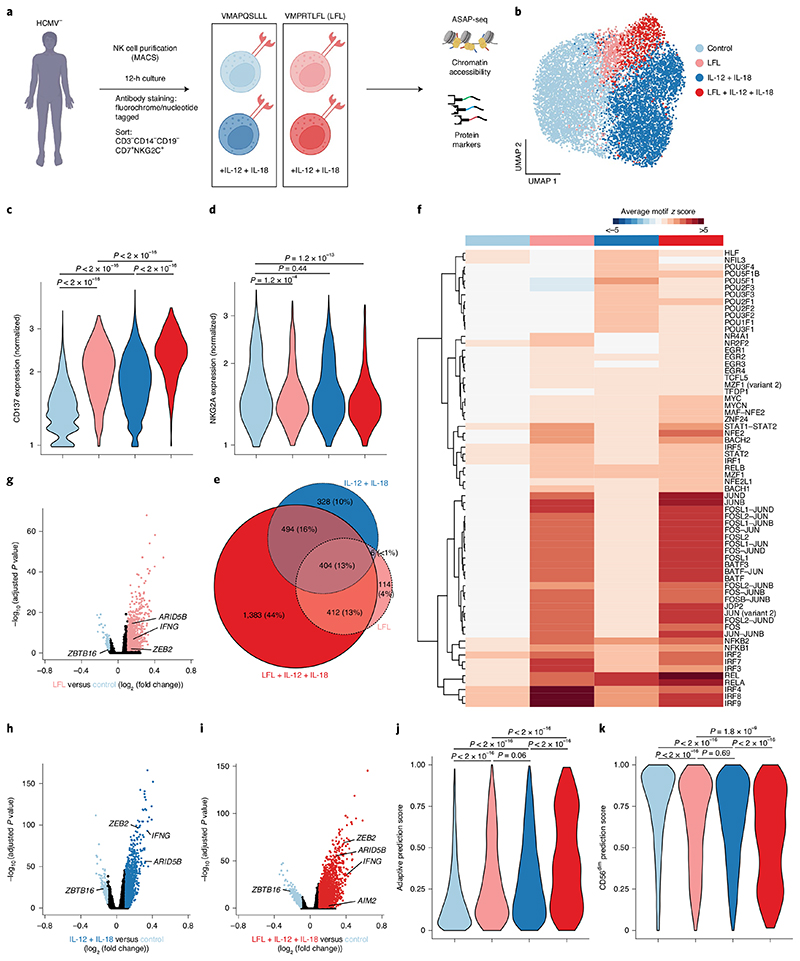
Synergistic imprinting by HCMV peptides and proinflammatory cytokines. **a**, NK cells from two HCMV^−^ individuals were cocultured for 12 h with RMA-S/HLA-E target cells pulsed with the indicated peptides and 10 ng ml^−1^ IL-15 in the presence (dark shades) or absence (light shades) of IL-12 and IL-18. Different conditions were marked with nucleotide-labeled hashtags and were analyzed by ASAP-seq. **b**, UMAP embedding, clusters were annotated based on clear enrichment of cells from the indicated conditions. **c**,**d**, Surface expression of CD137 (**c**) and NKG2A (**d**) per cluster; data were analyzed by two-sided Wilcoxon rank-sum test with a Benjamini–Hochberg adjustment. **e**, Euler diagram illustrating overlap of DARs between clusters. **f**, Motif activity of differentially active motifs represented as average chromVAR deviation scores per cluster. **g**–**i**, DARs between LFL and control (**g**), IL-12 + IL-18 and control (**h**) and LFL + IL-12 + IL-18 and control (**i**) by logistic regression. **j**,**k**, Prediction scores for classification as adaptive (**j**) and CD56^dim^ (**k**) NK cells based on integration with the ex vivo dataset as reference; data were analyzed by two-sided Wilcoxon rank-sum test with a Benjamini-Hochberg adjustment. Created with BioRender.com.

Activation by interleukin-12 (IL-12) and IL-18 in combination with engagement of NKG2C by HCMV-derived peptides induces the specific expansion of NKG2C^+^ NK cells with some of the transcriptional characteristics also observed ex vivo^10^. To assess whether these signals drive epigenetic reprogramming, including AP1 TF activity, and mimic HCMV-induced activation of naive NKG2C^+^ NK cells, we cocultured NK cells from HCMV^−^ donors for 12 h with IL-15 and RMA-S/HLA-E target cells pulsed with the NKG2C-activating VMAPRTLFL (LFL) or non-activating VMAPQSLLL peptide in the presence or absence of IL-12 and IL-18. We marked the different conditions with individual nucleotide-barcoded hashtags, sorted for NKG2C^+^ cells and performed ASAP-seq (Fig. 4a).

**Extended Data Fig. 5:**
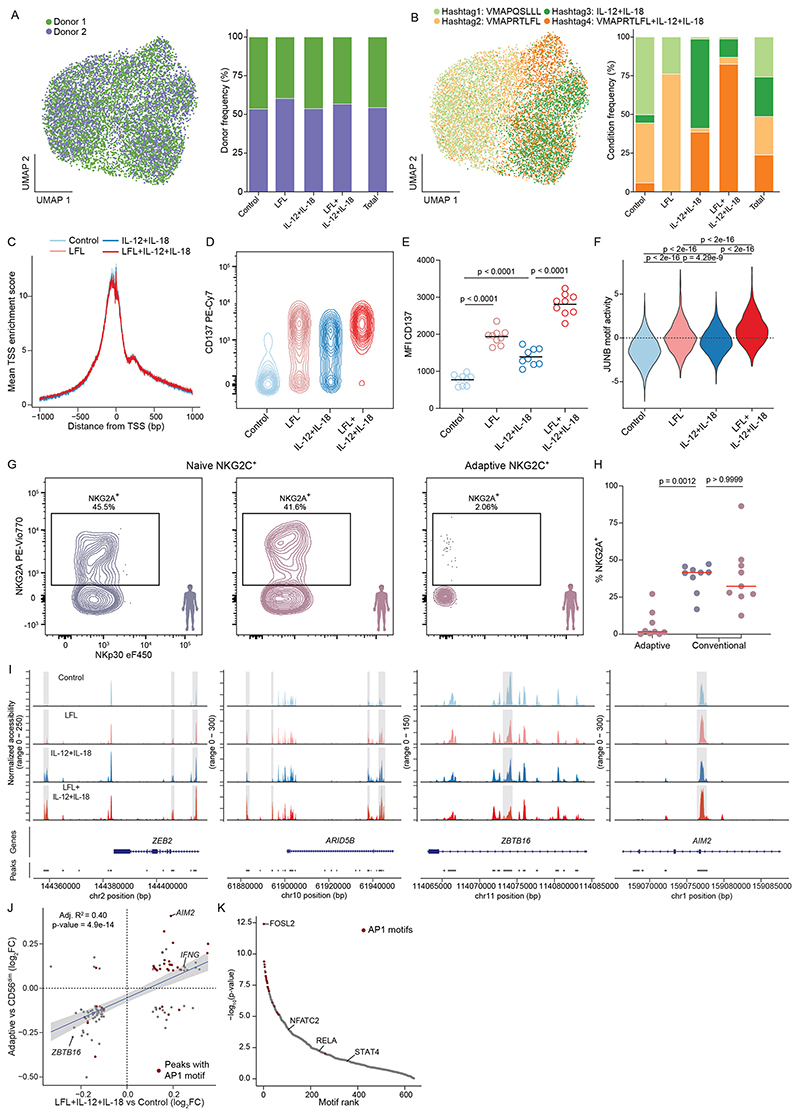
Synergistic imprinting by HCMV peptides and pro-inflammatory cytokines. **(a)** Distribution of cells colored by donor origin and quantification per cluster. **(b)** Distribution of cells colored by culture condition and quantification per cluster, utilized for cluster annotation. **(c)** Transcription start site (TSS) enrichment plot overlaid for all clusters. **(d** + **e)** Representative (D) and mean (E) CD137 expression on NKG2C^+^ NKG2A^−^ NK cells from HCMV^−^ donors (n = 9) cultured under the indicated conditions; One-way ANOVA with Holm-Šídák adjustment. **(f)** JUNB motif activity per cluster; Wilcoxon rank sum test with Benjamini-Hochberg adjustment. **(g-h)** Representative (G) and quantification (H) of NKG2A expression in naïve and adaptive NKG2C^+^ NK cells from HCMV^−^ (n= 9) and HCMV^+^ (n = 9) donors; see Extended Data Fig. 2g for gating strategy; Kruskal-Wallis test with Dunn’s post-hoc test. **(i)** Accessibility of regions near key adaptive NK cell-related genes regulated after stimulation. **(j)** Linear correlation between differentially accessible regions both induced by *in vitro* activation and observed *ex vivo* between adaptive and CD56^dim^ NK cells; error bands show 95 % confidence interval, p-value from two-sided F-test. **(k)** Motif enrichment in open chromatin regions displayed in (J) as determined by one-sided hypergeometric test with Benjamini–Hochberg adjustment. Created with BioRender.com.

Analysis of high-quality cells clustering by condition and irrespective of donors (Methods) enabled us to identify the epigenetic signatures acquired by activated NKG2C^+^ NK cells in a stimulus-dependent fashion, defining four main clusters clearly enriched for cells cultured under the respective conditions, that is, non-activated (‘control’), activated by LFL peptide (‘LFL’), proinflammatory cytokines (‘IL-12 + IL-18’) or a combination thereof (‘LFL + IL-12 + IL-18’; Fig. 4b, Extended Data Fig. 5a,b and Methods). The resulting chromatin accessibility profiles had a similar TSS enrichment (Extended Data Fig. 5c) as observed ex vivo (Extended Data Fig. 1b), irrespective of activation. Activation status of cells recruited in stimuli-associated clusters was exemplified by surface upregulation of CD137 (4-1BB), with maximal expression observed on cells receiving both peptide and cytokine stimulation (Fig. 4c). Differences in expression levels of CD137 across conditions were confirmed by flow cytometry (Extended Data Fig. 5d,e), validating the proteogenomic-based cluster annotations.

While IL-12 + IL-18 stimulation alone promoted the activation of NKG2A^+^NKG2C^+^ naive NK cells, hardly any NKG2A^+^NKG2C^+^ cells were recruited into response patterns induced by peptide stimulation (Fig. 4d), in line with the dominant inhibitory function of NKG2A engagement^33^. As, in contrast to adaptive NKG2C^+^ NK cells^26^, a large fraction of naive NKG2C^+^ NK cells coexpress NKG2A (Extended Data Fig. 5g,h), these data suggest that peptide recognition might be decisive in biasing the pool of naive NKG2C^+^ cells recruited during HCMV infection toward NKG2A^−^NKG2C^+^ cells.

Activation via proinflammatory cytokines or by peptide recognition via NKG2C induced pronounced chromatin remodeling, manifesting in 1,232 and 936 differentially accessible regions (DARs), respectively, compared to the control condition (Fig. 4e). Most regions underwent remodeling only after synergistic activation by the two combined stimuli (2,693 DARs; Fig. 4e), with different TF families contributing to these effects (Fig. 4f). Besides IRFs, EGR, MYC and NF-κB, LFL peptide activation induced remarkable activity of AP1-family TFs. Consistent with reports in mice^34^, proinflammatory cytokines IL-12 and IL-18 were highly specific in their induction of POU TF-family activity, along with NF-κB. However, clear synergism between cytokines and peptide became apparent also on the TF motif activity level, in particular for AP1 (Fig. 4f and Extended Data Fig. 5f). Importantly, the two stimuli induced several features associated with adaptive NK cells ex vivo. This included opening of chromatin in the *ZEB2* and *ARID5B* loci, which was mainly dependent on proinflammatory cytokines or similarly enabled by each individual stimulus, respectively, as well as closing of the *ZBTB16* locus (Fig. 4g–i and Extended Data Fig. 5i). However, most of these changes were more pronounced after synergistic activation, as was also the case for chromatin accessibility in the *AIM2* region (Fig. 4g–i and Extended Data Fig. 5i). To globally assess which of the activation-induced signatures best reconciles the adaptive signature, we performed anchor-based integration of the in vitro stimulated cells with our ex vivo dataset (Methods)^35^, thereby enabling a classification of the in vitro induced signatures based on chromatin profiles observed ex vivo. The highest adaptive prediction score was observed for cells activated by both stimuli, in concert with a reduction of the prediction score for conventional CD56^dim^ NK cells (Fig. 4j,k). The close relatedness of the adaptive ex vivo signature to the chromatin remodeling induced by synergistic in vitro activation was further supported by a strong correlation of overlapping DARs when comparing CD56^dim^ to adaptive NK cells and when comparing control to LFL + IL-12 + IL-18 activated cells (Extended Data Fig. 5j). Among these conserved DARs, we identified core features of adaptive NK cells, such as reduced accessibility at the *ZBTB16* locus and increased accessibility of *AIM2* or *IFNG* loci (Extended Data Fig. 5j). Importantly, these modulated chromatin regions showed a strong enrichment of AP1 motifs, whereas NF-κB, NFAT and STAT4 motifs were much less enriched (Extended Data Fig. 5k). Together, these findings directly connect proinflammatory cytokines and NKG2C-activating peptide, both provided during HCMV infection, to the characteristic phenotype and epigenetic remodeling of adaptive NK cells as observed ex vivo. Their synergism manifested on several layers from surface upregulation of CD137, over TF motif activity, to global chromatin remodeling, thereby recapitulating features of adaptive NK cell signatures including open chromatin enriched for AP1 motifs.

### Convergent and divergent epigenetic features of adaptive NK cells

**Extended Data Fig. 6:**
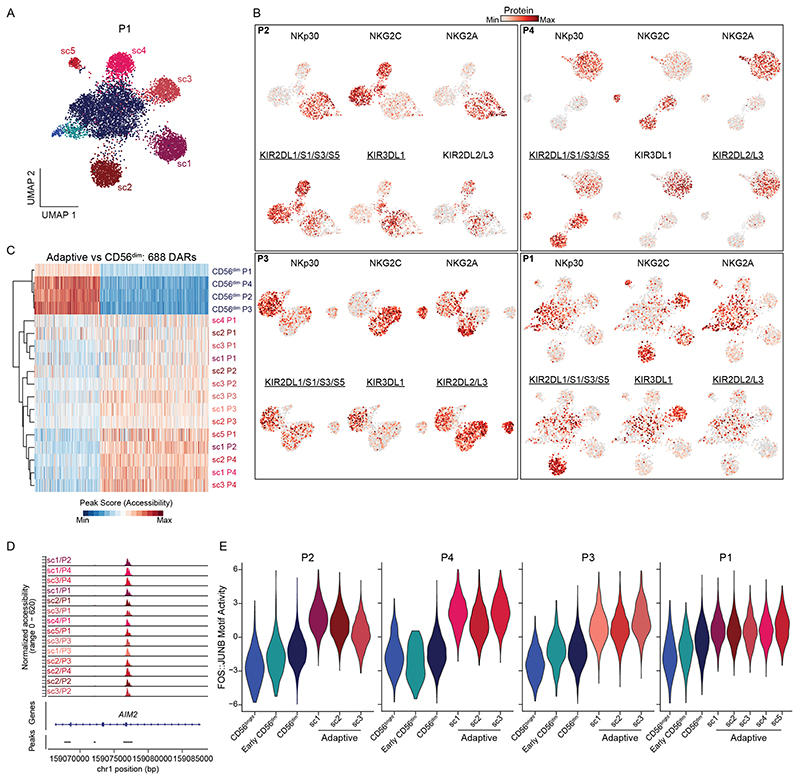
Convergent and divergent epigenetic features of adaptive NK cells. **(a)** UMAP embedding of NK cells from HCMV^+^ individual P1 analyzed by scATAC-seq. **(b)** Surface expression of the indicated proteins projected on the UMAP embeddings for each donor. Educating KIRs are underlined, see Supplementary Table 2. **(c)** Column-scaled accessibility of differentially accessible regions (DARs) shared between at least three donors comparing the total adaptive and CD56^dim^ compartments. **(d)** Accessibility of adaptive NK-cell defining *AIM2* region within each donor and adaptive subcluster. **(e)** FOS::JUNB motif activity within each donor. **(f)** Accessibility of representative subcluster-specific chromatin regions for each donor.

**Extended Data Fig. 7:**
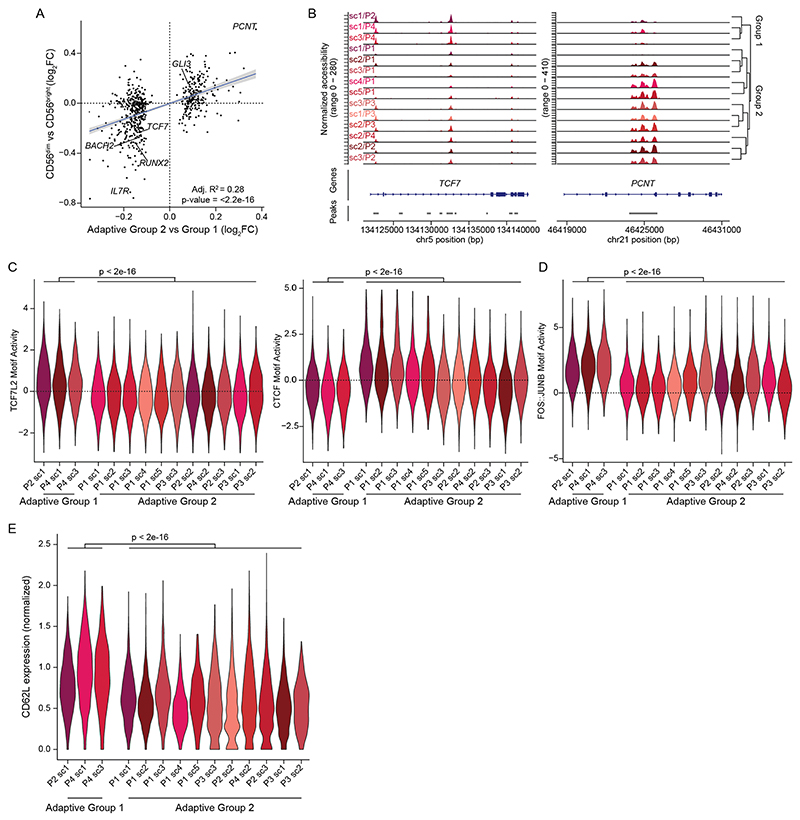
Adaptive subcluster groups are defined by a maturation gradient. **(a)** Linear correlation between DARs comparing the two adaptive subcluster groups and CD56^dim^ to early CD56^dim^ NK cells; error bands show 95 % confidence interval, p-value from two-sided F-test. **(b)** Accessibility of adaptive group-defining regions within *TCF7* and *PCNT* for each donor and adaptive subcluster. **(c)** TCF7L2 and CTCF motif activity for each donor and adaptive subcluster; two-sided Wilcoxon test with Bonferroni adjustment. **(d)** FOS::JUNB motif activity for each donor and adaptive subcluster; two-sided Wilcoxon test with Bonferroni adjustment. **(e)** Normalized CD62L surface expression per cluster; two-sided Wilcoxon rank sum test with Bonferroni adjustment.

**Fig. 5:**
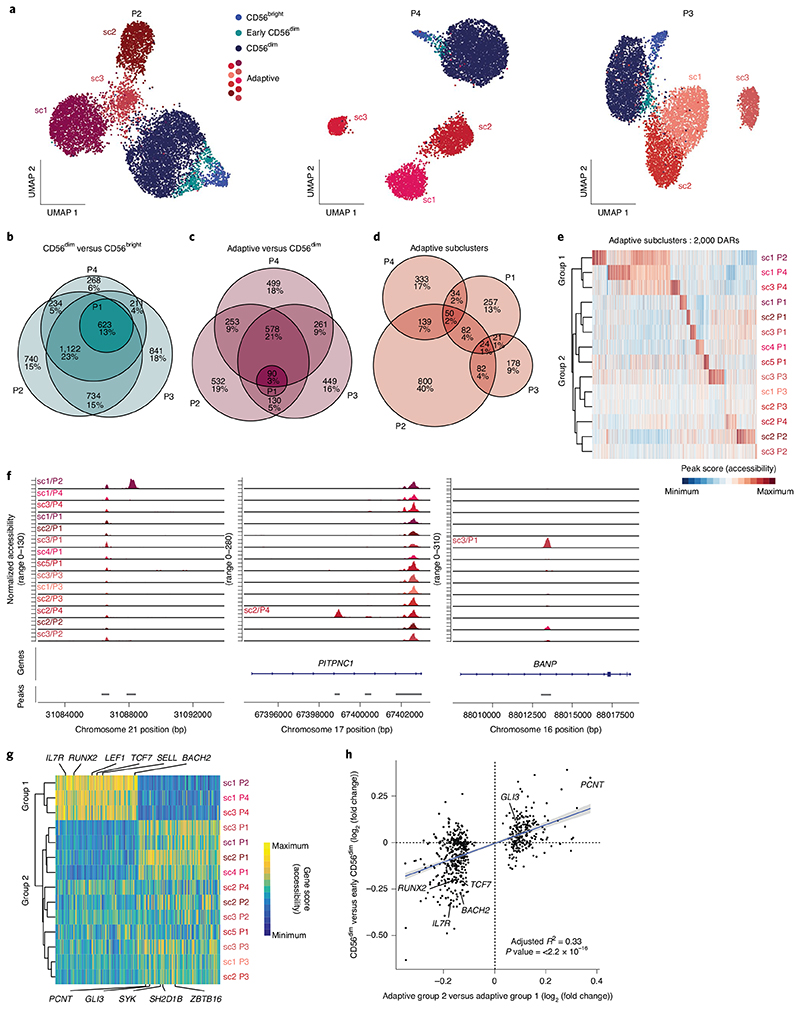
Convergent and divergent epigenetic features of adaptive NK cells. **a**, UMAP embedding of NK cells from three HCMV^+^ individuals analyzed by scATAC-seq; sc, subcluster. **b**–**d**, Euler diagrams illustrating the overlap of DARs across donors comparing CD56^dim^ and CD56^bright^ (**b**), adaptive and CD56^dim^ (**c**) or adaptive subclusters (**d**). **e**, Column-scaled accessibility of subcluster-defining DARs and hierarchical clustering of adaptive subclusters for all individuals. **f**, Accessibility of representative subcluster-specific chromatin regions for each donor; bp, base pairs. **g**, Top 100 differential column-scaled gene scores for each adaptive subcluster group by Wilcoxon rank-sum test. **h**, Linear correlation between DARs comparing the two adaptive subcluster groups and CD56^dim^ to early CD56^dim^ NK cells; error bands show 95% confidence interval. The *P* value was determined by two-sided *F*-test.

The integrated analysis of different HCMV^+^ donors revealed consistent epigenetic, transcriptional and phenotypic features of the adaptive NK cell signature ex vivo. However, integration of donors masked donor-specific heterogeneity within the adaptive NK cell compartment. Indeed, separate analysis of the epigenetic landscape of individual donors revealed clearly defined adaptive subclusters (Fig. 5a and Extended Data Fig. 6a). These populations were characterized by an adaptive phenotype, largely lacking surface expression of NKp30 and NGK2A, and were mostly positive for NKG2C and self-MHC-specific KIRs (Extended Data Fig. 6b and Supplementary Table 2). Interestingly, subcluster 4 in donor P1 likely represents an NKG2C^−^ adaptive NK cell expansion (Extended Data Fig. 6b), as previously described by others^11,36,37^. To assess the degree of convergence of the adaptive NK cell signature, we analyzed the overlap of DARs between the total adaptive and the CD56^dim^ compartment across HCMV^+^ donors (P1, P2, P3 and P4), revealing that around half of all DARs were shared between at least three donors, similar to the signatures distinguishing CD56^bright^ from CD56^dim^ NK cells (Fig. 5b,c). Consistent with integration into a donor-overarching adaptive cluster (Fig. 1b), this adaptive signature was shared by all adaptive subclusters, separating them from the conventional CD56^dim^ populations (Extended Data Fig. 6c). Many of the features identified in the integrated analysis were highly penetrant across donors and subclusters, such as the peak in the proximity of *AIM2* (Extended Data Fig. 6d) and the strongly enhanced AP1 motif activity (Extended Data Fig. 6e), further supporting the idea that this convergent epigenetic signature reflects a coordinated program induced in response to signals received during HCMV infection. In contrast to these shared aspects, DARs between adaptive subclusters were much less conserved (Fig. 5d). Each adaptive subcluster showcased highly unique open chromatin regions (Fig. 5e,f), underlining their distinctive epigenetic makeup. Nevertheless, hierarchical clustering divided the adaptive subclusters into two groups, mainly driven by overlapping signatures of three subclusters (Fig. 5e). Importantly, when directly comparing the gene scores of the two groups, two clear signatures emerged, which had an intriguing overlap with those defining CD56^bright^/early CD56^dim^ NK cells, such as *RUNX2, BACH2, TCF7, IL7R* and *SELL* (encoding CD62L), on the one end and CD56^dim^-associated genes, such as *GLI3* or *ZBTB16*, on the other end (Fig. 5g). Indeed, differential accessibility of the respective chromatin regions between the adaptive subcluster groups correlated significantly with the differences between CD56^bright^/early CD56^dim^ and CD56^dim^ NK cells (Fig. 5h and Extended Data Fig. 7a) and followed consistent patterns as exemplified for chromatin regions in the proximity of *TCF7* or *PCNT* (Extended Data Fig. 7b), suggesting that conventional NK cell maturation signatures are preserved after adaptive differentiation. This was also supported by differential activity of CD56^bright^/ early CD56^dim^-associated motifs, such as TCF7L2, on the one hand and the CD56^dim^-associated CTCF motif on the other hand (Extended Data Fig. 7c). Strikingly, the group resembling CD56^bright^/early CD56^dim^ NK cells was also marked by surface expression of CD62L (Extended Data Fig. 7e), consistent with its expression by CD56^bright^ and early CD56^dim^ NK cells within the conventional compartment^38^ (Fig. 1c,e). Additionally, the clusters that shared part of the CD56^bright^/early CD56^dim^ signature had relatively higher AP1 motif activity (Extended Data Fig. 7d), suggesting that the two groups might also reflect a different extent of adaptive chromatin remodeling. Overall, this donor-centric analysis revealed an unappreciated heterogeneity within the adaptive NK cell compartment defined by a highly diverse set of unique, subcluster-specific peaks and signatures resembling gradients in conventional NK cell maturation, possibly reflecting the cell of origin.

### Clonal expansion underlies divergent signatures of adaptive NK cells

**Extended Data Fig. 8:**
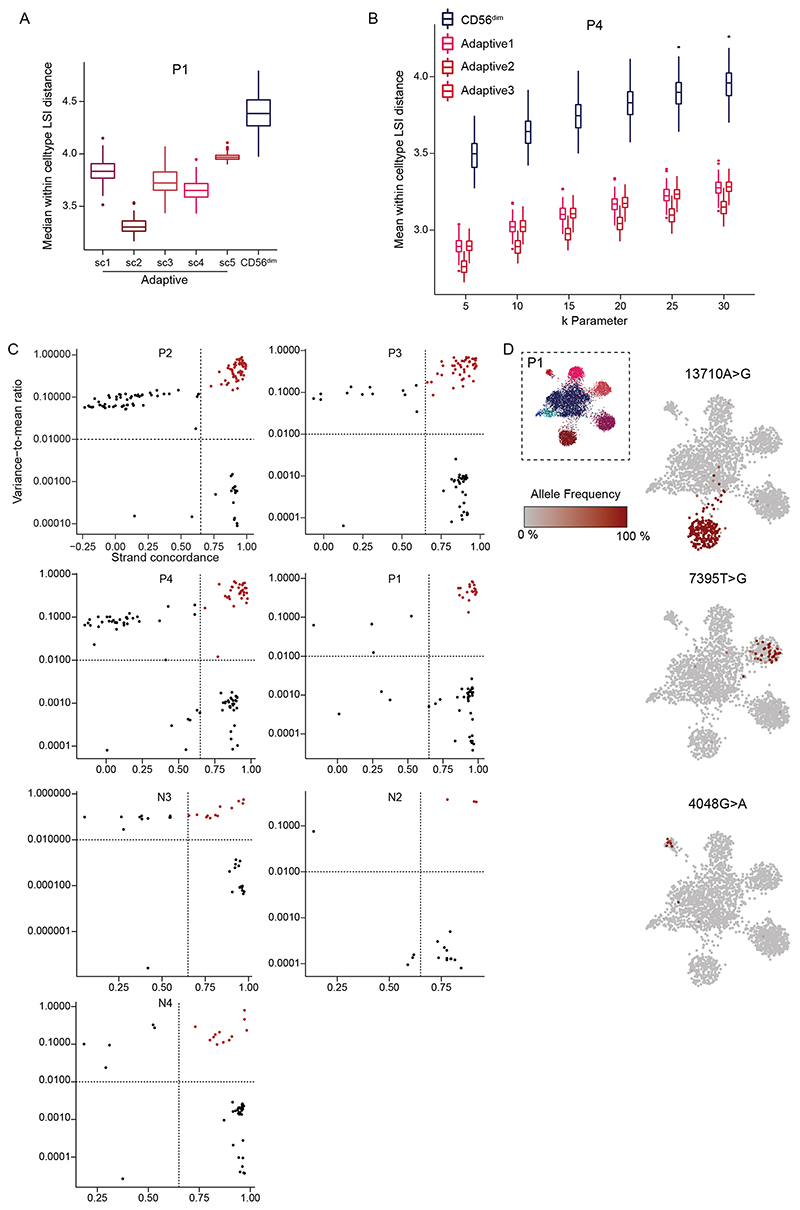
Clonal expansion underlies divergent epigenetic signatures of adaptive NKG2C^+^ NK cells. **(a)** Cluster heterogeneity of donor P1 NK cells as assessed by measuring the median distance of 200 randomly sampled cells to their k nearest neighbors and repeating this process 100 times. **(b)** The k parameter was varied from 5-30 for donor P4 to assess robustness of this heterogeneity metric. The upper and lower hinges of boxplots correspond to the first and third quartiles, respectively. The upper and lower whiskers extend to the largest/smallest value no further than 1.5 times the interquartile range from the hinges. Outliers beyond whiskers are displayed as individual points. **(c)** Strandconcordance and variance-to-mean ratio applied for extraction of informative mutations for each donor. **(d)** Allele frequency of representative somatic mtDNA mutations projected onto UMAP embedding for donor P1.

**Extended Data Fig. 9:**
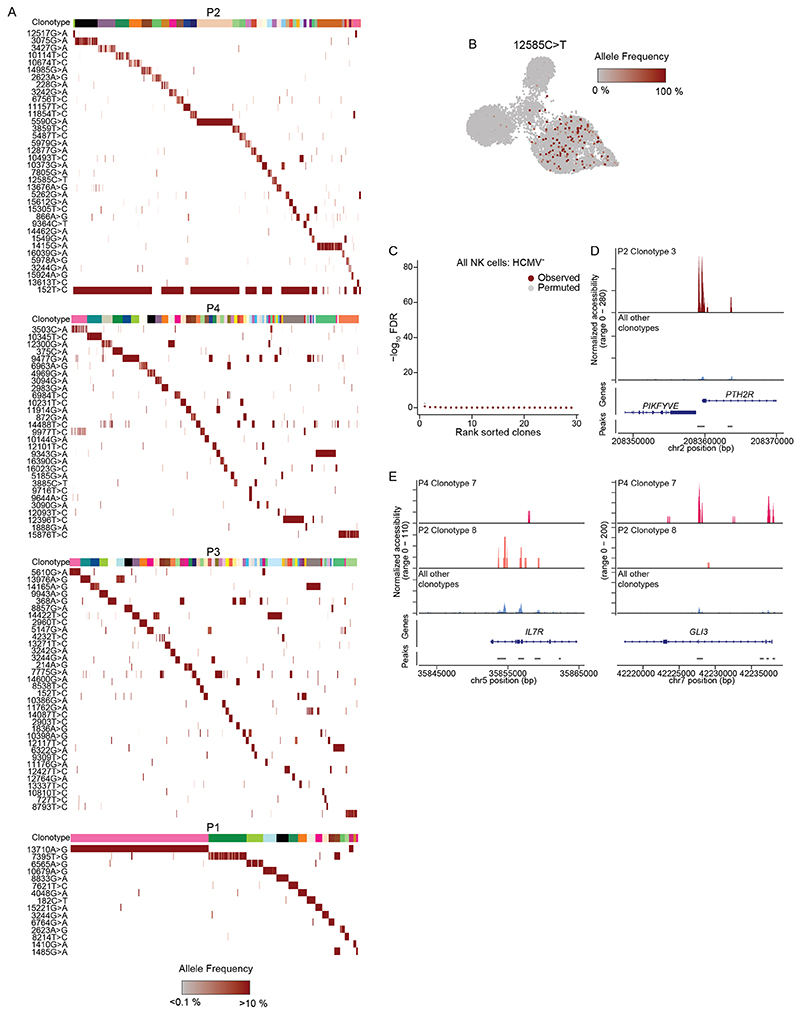
Adaptive clonotypes are associated to unique epigenetic identities. **(A)** Clonotypes defined by clustering on per-cell allele frequency for all high-confidence variants. **(b)** Allele frequency of representative somatic mtDNA mutation within the conventional compartment of donor P2. **(c)** Association of clonotypes to clusters defined by chromatin accessibility for all NK cells from HCMV^−^ donors (n = 3). **(d-e)** Representative open chromatin regions specifically associated to individual clonotypes.

**Fig. 6:**
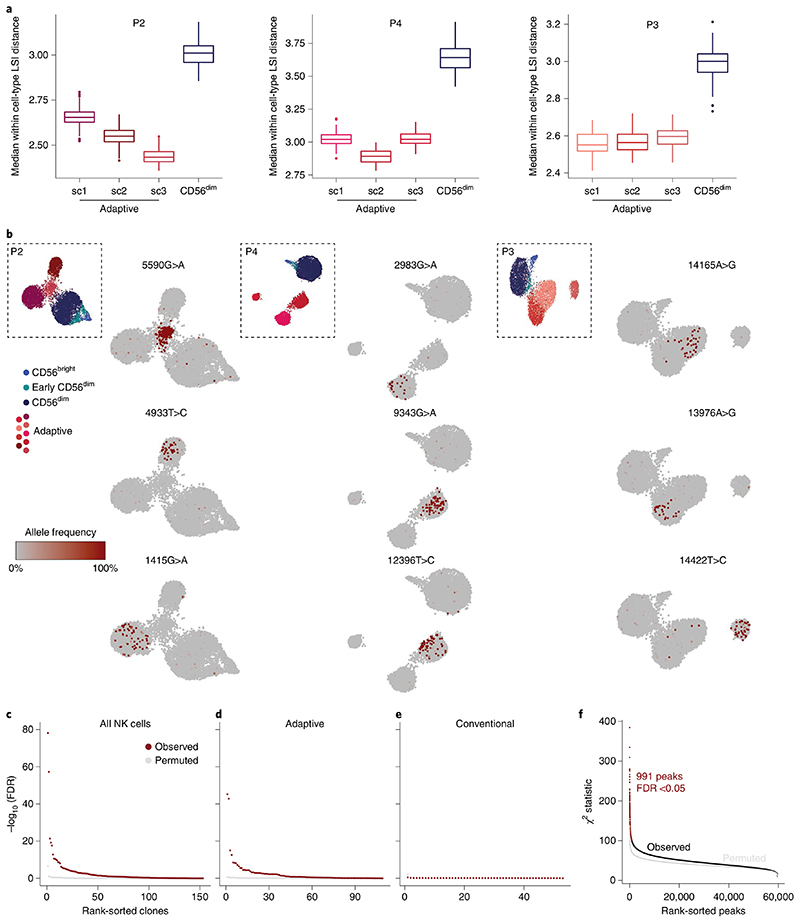
Clonal expansion underlies divergent epigenetic signatures of adaptive NK cells. **a**, Cluster heterogeneity as assessed by measuring the median distance of 200 randomly sampled cells to their 10 nearest neighbors and repeating this process 100 times. The upper and lower hinges of the box plots correspond to the first and third quartiles, respectively. The upper and lower whiskers extend to the largest/smallest value no further than 1.5× the interquartile range from the hinges. Outliers beyond the whiskers are displayed as individual points. **b**, Allele frequency of representative somatic mtDNA mutations projected onto UMAP embedding for each HCMV^+^ donor. **c**–**e**, Association of clonotypes to clusters defined by chromatin accessibility for all NK cells (**c**), adaptive NK cells (**d**) and conventional NK cells (**e**); false discovery rate (FDR) from χ^2^ tests for the observed and randomly permuted clonotype–cluster relationships for all donors. **f**, Association of clonotypes to open chromatin regions as assessed by χ^2^ test for the observed and randomly permuted clonotype–peak relationships.

In contrast to the convergent remodeling at key adaptive genes and a pronounced enrichment of AP1 motifs, the large number of unique peaks detected only in individual adaptive subclusters represents a diversification that is difficult to reconcile with a coordinated differentiation program. We hypothesized that this apparent diversification might result from the expansion of individual naive NKG2C^+^ cells with unique accessible chromatin regions. Expansion of rare founder cells would amplify these regions beyond the detection threshold while at the same time reduce the epigenetic heterogeneity of their progeny. Hence, as a measure for cluster heterogeneity, we analyzed the average distance between the *k* nearest neighbors within the latent semantic indexing (LSI) space representing their chromatin accessibility landscape (Methods). Notably, all adaptive subclusters were less heterogeneous than their corresponding conventional CD56^dim^ compartment, as demonstrated by a uniformly lower average distance (Fig. 6a and Extended Data Fig. 8a). This heterogeneity measure was robust over a wide range of values for *k* (Extended Data Fig. 8b), indicating a focused epigenetic profile of individual adaptive NK cell clusters, potentially resulting from a selection bottleneck during recruitment, followed by clonal expansion. To test the hypothesis that divergent epigenetic profiles of adaptive subclusters are associated with clonal expansions, we applied a recently published method that exploits mtDNA mutations as endogenous barcodes to reconstruct clonal relationships and concomitantly links them to epigenetic and cell surface phenotypes^16–18^. Applying this method to four HCMV^+^ and three HCMV^−^ donors, we identified informative mtDNA mutations by high strand concordance and variance-to-mean ratio (Extended Data Fig. 8c). In the HCMV^+^ donors, many of these mutations were specifically enriched in the adaptive NK cell compartment (Fig. 6b and Extended Data Fig. 8d). Importantly, clonotypes defined by individual mutations (Extended Data Fig. 9a and Methods) were significantly associated not only with adaptive NK cells as a whole but also with specific subclusters, as demonstrated by the χ^2^ statistic of the observed compared to randomly permuted clonotype–cluster relationship (Fig. 6c,d). This striking concordance between mitochondrial mutations and epigenetic identities reveals the inheritance of epigenetic states as a defining clonal mark. Conversely, such an association was absent in the conventional NK cell compartment (Fig. 6e and Extended Data Fig. 9b), suggesting that mitochondrial mutations of conventional NK cells do not flag clonal expansions but might instead be present already on the progenitor level. Similarly, there was no significant association between clonotypes and epigenetic identities in HCMV^−^ donors (Extended Data Fig. 9c), underlining the specific clonal expansion of NKG2C^+^ NK cells in response to HCMV. Donor-specific patterns further highlighted the degree of clonal expansion of the adaptive subclusters. For example, a large fraction of cells in subcluster 3 of donor P2 carried the 5590G>A mutation at near homoplasmic frequencies (Fig. 6b), coinciding with expression of a second self-MHC-specific KIR3DL1, which was absent from the other adaptive subclusters in this individual (Extended Data Fig. 6b).

Finally, association of clonotypes to individual subclusters defined by chromatin accessibility highlighted the epigenetic similarity of cells belonging to one clonotype. This was further supported by the significant association between individual open chromatin regions and clonotypes (Fig. 6f). Importantly, we found regions that were uniquely accessible in individual donors and clonotypes (Extended Data Fig. 9d), while a part of this diversity was again driven by the opposing accessibility in the proximity of genes associated with conventional NK cell maturation, such as *IL7R* and *GLI3* (Extended Data Fig. 9e), suggesting that these signatures are indeed clonally inherited. Together, we demonstrate that subclusters of adaptive NK cells contain individual clonotypes with private chromatin accessibility profiles, strongly supporting clonal expansion as a driving force of adaptive NK cell generation and epigenetic diversification.

### Adaptive NK cell clonotypes are stably maintained over time

**Extended Data Fig. 10:**
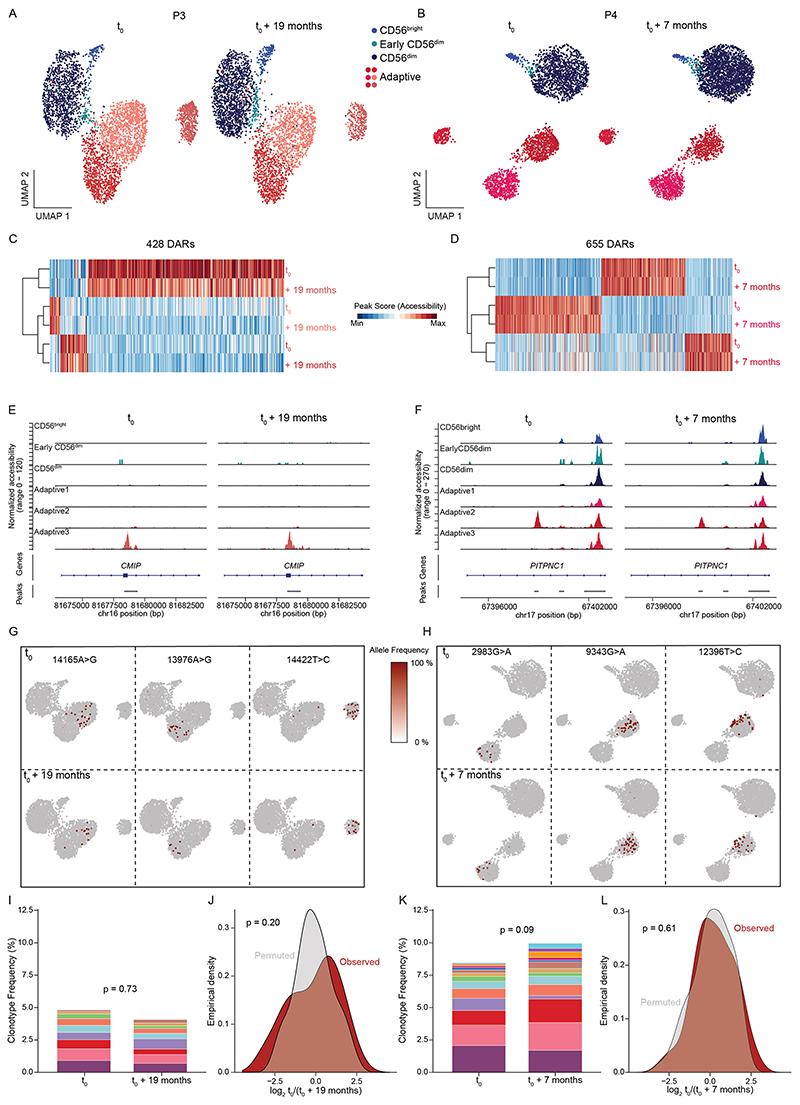
Adaptive NK cell clonotypes are stably maintained over time. **(a)** UMAP embedding of NK cells from HCMV^+^ donor P3 and P4 analyzed by scATAC-seq at two different time points. **(c-f)** Stability of overall (C-D) and representative (E-F) subcluster-defining differentially accessible regions (DARs) over time; column-scaled. **(g-h)** Representative clonotype-defining mutations projected onto UMAP embeddings at the two time points. **(i** + **k)** Clonotype frequency of adaptive NK cell clonotypes within total adaptive NK cell compartment over time; Fisher’s exact test with Monte Carlo simulation. **(j** + **l)** Observed and permuted distribution of clonotype log_2_ fold-changes between time points; two-sided Kolmogorov-Smirnov test.

**Fig. 7:**
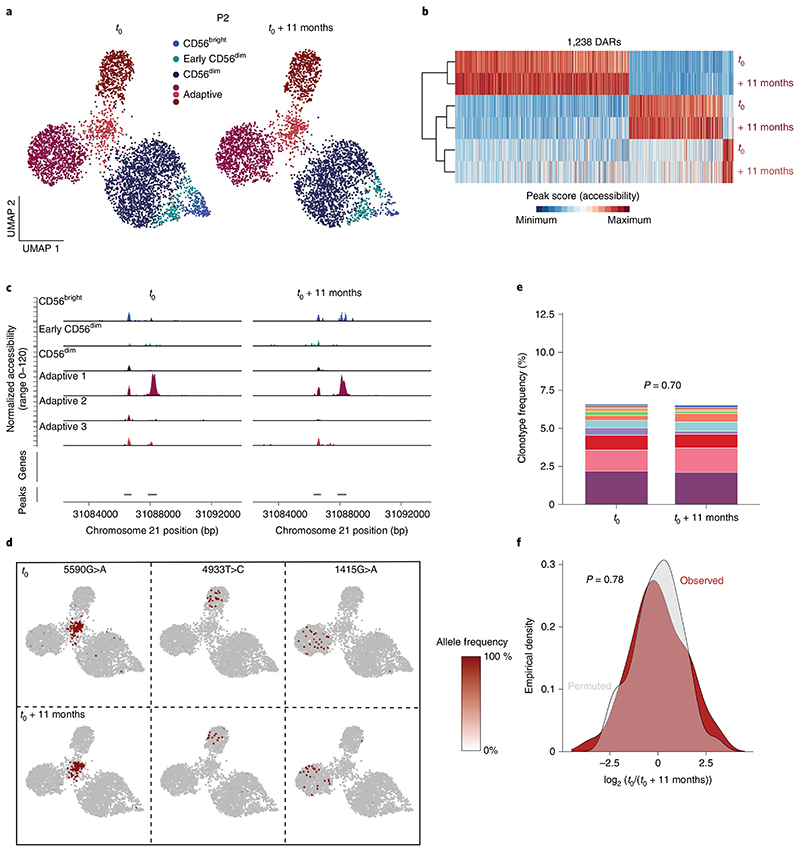
Adaptive NK cell clonotypes are stably maintained over time. **a**, UMAP embedding of NK cells from HCMV^+^ donor P2 analyzed by scATAC-seq at two different time points. **b**,**c**, Stability of overall (**b**) and representative (**c**) subcluster-defining DARs over time; data are column scaled. **d**, Representative clonotype-defining mutations projected onto UMAP embeddings at the two time points. **e**, Clonotype frequency of adaptive NK cell clonotypes within the total adaptive NK cell compartment over time; data were analyzed by Fisher’s exact test with Monte Carlo simulation. **f**, Observed and permuted distribution of clonotype log_2_ (fold change) values between time points; data were analyzed by two-sided Kolmogorov–Smirnov test.

The specific association of non-overlapping clonotypes to adaptive subclusters suggests a degree of clonal and epigenetic stability. To test this hypothesis, we performed a longitudinal follow-up analysis of three HCMV^+^ donors after 11, 7 and 19 months, respectively. Clusters defined by chromatin accessibility were unchanged between the two time points, supporting stable maintenance of the frequency and composition of the adaptive NK cell pool down to the subcluster level (Fig. 7a and Extended Data Fig. 10a,b). Further, the subcluster-specific open chromatin regions remained stable in this time frame (Fig. 7b,c and Extended Data Fig. 10c–f). Importantly, the same clonotype-defining mutations were detected at both time points and remained associated with the original subclusters (Fig. 7d and Extended Data Fig. 10g,h), highlighting their persistence and demonstrating that adaptive subclusters represent independent and stably maintained clonal expansions. Along these lines, even frequencies of clonotypes significantly associated with the adaptive compartment were stably maintained over time (Fig. 7e and Extended Data Fig. 10i,k), which was further supported by an unskewed total clonotype distribution across time points (Fig. 7f and Extended Data Fig. 10j,l). Overall, the longitudinal follow-up demonstrates the long-term clonal maintenance of adaptive NK cells with stable chromatin accessibility profiles in progeny arising from individual clonal founders, which are key features of ‘epigenetic memory’, as previously ascribed to the adaptive immune system.

## Discussion

Previous studies have revealed conserved signatures of adaptive NK cells driven by coordinated changes in their gene regulatory networks, such as downregulation of the TF PLZF^36^ or increased activity of the T cell lineage TF BCL11B^24^. Conversely, insights into their heterogeneity have remained limited to the diversification of signaling adaptor expression between and within individuals^36^. Here, we provide an integrated, multiomic single-cell analysis of human NK cells, enabling us to study both convergent and divergent aspects of adaptive NK cell biology.

Our finding of a strong enrichment of AP1 motifs in adaptive NK cell chromatin extends observations made in different cell types^4,31,39–42^, further supporting the concept of a conserved inflammatory memory signature imprinted by AP1 TFs^4,32^. Here, we demonstrate that HCMV infection can induce this characteristic inflammatory memory in human NK cells, revealing a surprising conservation of this signature across stimuli and species. Consistent with reports in mice^43,44^ and our own studies in humans^10^ we demonstrate that synergistic activation of naive NGK2C^+^ NK cells via CD94/NKG2C and proinflammatory cytokines most closely recapitulated the adaptive NK cell signature observed ex vivo and induced the most pronounced activity of AP1 TFs.

Conversely, our donor-specific analysis revealed two different layers of adaptive NK cell heterogeneity. First, we identified two underlying signatures conserved across adaptive NK cells from HCMV^+^ donors, which resembled maturation differences within the conventional NK cell compartment. Second, we unveiled unique, donor-specific, that is, private, open chromatin regions characterizing individual subclusters. Based on the high degree of clonality of adaptive NK cell expansions, we propose that both of these levels of heterogeneity can be explained by clonal inheritance of epigenetic traits, enabling an epigenetic founder effect. Acting as a clonal bottleneck, the recruitment of individual NK cells into the adaptive response and their massive expansion might skew the distribution of open chromatin regions. Hence, the two layers of adaptive NK cell heterogeneity likely reflect the degree of diversity and maturation stage within the original naive NKG2C^+^ pool before acquisition of the superimposed adaptive differentiation program. Together with our observation that naive NKG2C^+^NK cells span the whole spectrum of conventional NK cell maturation, these data suggest that naive NKG2C^+^ NK cells might be recruited into the adaptive response at these different stages and preserve a part of their initial epigenetic identity. This is consistent with findings in mice, where pre-existing NK cell subtypes were preserved after murine CMV-induced expansion^45^. Alternatively, these might represent different stages of adaptive NK cells, although the association of clonotypes with individual subclusters and their stability in time argue against this interpretation. Regarding the unique open chromatin regions associated with adaptive NK cell subclusters, we envisage that they become evident due to the drastic expansion of individual NK cell clones, which at the same time underlies the reduced heterogeneity of adaptive subpopulations. Consequently, we propose that the macroscopic picture of epigenetic diversification, manifesting, for example, in variable expression of signaling adaptors^36^, is created by the coexistence of several clonal expansions that are, in fact, epigenetically focused.

Importantly, our findings enable us to generalize some of the mechanisms driving clonal expansion and their consequences within the immune system as a whole. One such unifying prerequisite for clonal expansion is the competitive recruitment of a limited number of cells to emerge as clones from a diverse population. Despite their reliance on a conserved receptor for HCMV recognition, other cell-intrinsic features of NK cells are strongly diversified, especially their combined cell surface receptor expression profiles^46^, leading to selection of optimal receptor combinations. The low frequency of cells expressing NKG2C before HCMV infection is the first limitation of the original pool activated by HCMV peptides. Further, in response to peptide and cytokine stimulation, only NKG2A^−^ NK cells underwent remodeling that resembled adaptive NK cells the closest, suggesting that NKG2A expression further limits the pool of naive NKG2C^+^ cells optimally responding to HCMV. Expression levels of NKG2C might also contribute to selection, as adaptive NKG2C^+^ NK cells express higher levels of NKG2C than their naive counterparts^47^, and a similar mechanism occurs for Ly49H^+^ NK cells in mice^12,48^. Clonal selection can further be influenced by complex biological traits that are heterogeneously distributed among cells before or after activation, such as proliferation and differentiation kinetics^49,50^ or metabolic capacity^51^. More studies will be needed to distinguish the adaptive NK cell features modulated by external signals from those conferring a selection advantage within the naive repertoire and the ones that are stochastic passengers of the clonal expansion process. The potential of non-genetic inheritance mechanisms to impact cell fitness has recently been demonstrated in the context of tumor cell resistance against anticancer therapeutics^52^. As T cells can also clonally inherit transcriptional and epigenetic diversity^53^, these analyses might provide general insights into the drivers of clonal success in different cell types independent of receptor specificity.

Finally, prolonged survival of adaptive NK cells has been postulated based on the observation of stable expression of KIR and signaling adaptor patterns^36^, and evidence from individuals with mutations in *GATA2* or *PIGA* supports this concept^54,55^. Here, we demonstrated long-term persistence on the clonal level for at least 19 months, although the exact cellular mechanisms enabling clonal maintenance remain to be defined.

Here, we demonstrate the clonal expansion and long-term persistence of an innate immune population in a memory state in response to a naturally occurring infection and introduce clonal selection and epigenetic inheritance as mechanisms that shape human innate immune composition for optimized secondary responses. Understanding the mechanisms determining clonal fitness in adaptive and innate immune cells independent of diversified antigen receptors might open new avenues for fundamental research and therapeutic interventions.

## Online content

Any methods, additional references, Nature Research reporting summaries, source data, extended data, supplementary information, acknowledgements, peer review information; details of author contributions and competing interests; and statements of data and code availability are available at https://doi.org/10.1038/s41590-022-01327-7.

## Methods

### Human studies

All analyses were performed in compliance with the relevant ethical regulations, and all donors gave informed consent. Primary NK cells were isolated from freshly drawn peripheral blood of healthy donors (Supplementary Table 1) or from buffy coats obtained from Deutsches Rotes Kreuz (DRK) Blutspendedienst Nord-Ost. The Charité Ethics Committee approved the study (EA4/196/18 and EA4/059/17).

### Cell lines

RMA-S/HLA-E cells^56^ were maintained in complete medium (RPMI-1640 containing glutamine and supplemented with 10 % (vol/vol) fetal bovine serum (FBS), 20 μM β-mercaptoethanol and 100 U ml^−1^ penicillin–streptomycin; all Thermo Fisher) in the presence of 400 μg ml^−1^ hygromycin B (InvivoGen).

### Cell isolation

Peripheral blood mononuclear cells were isolated by density gradient centrifugation (Ficoll Paque Plus, GE Healthcare) and either processed immediately or cryopreserved in FBS containing 10% DMSO.

### HCMV serology

For buffy coats, CMV serology was performed at DRK Dresden. Serological status of fresh blood donors was analyzed by HCMV IgG enzyme-linked immunosorbent assay (IBL International) following the manufacturer’s instructions. All samples were measured in duplicate and were either clearly above or below the cutoff control.

### Flow cytometry

Cell suspensions were stained with combinations of the following fluorochrome-conjugated antibodies following established guidelines^57^: CD56 PE/Dazzle 594 (1:200), CD137 PE/cyanine7 (1:50) and CD57 BV605 (1:25; all BioLegend); CD7 BV786 (1:25), CD56 BUV737 (1:50), CD3 BUV805 (1:50), streptavidin BUV395 (1:100), CD16 BUV496 (1:50) and CD337 (NKp30) BV421 (1:25; all BD Biosciences); CD159a (NKG2A) biotin/PE-Vio770 (both 1:50) and CD159c (NKG2C) PE (1:100; all REAfinity, Miltenyi Biotec); CD337 (NKp30) eFluor 450 (1:25), CD3 APC-eFluor 780 (1:50), CD14 APC-eFluor 780 (1:50) and CD19 APC-eFluor 780 (1:50; all Thermo Fisher) and anti-FcεRI, γ subunit-FITC (1:50; Merck). Dead cells were excluded using Fixable Viability Dye eFluor 780 (Thermo Fisher) or a Zombie Aqua Fixable Viability kit (BioLegend). Data were acquired on an LSR Fortessa (BD Biosciences). Cell sorting was performed on a FACSAria II (BD Biosciences). FlowJo v10 was used for analysis of flow cytometry data, and GraphPad Prism v8.4.3 was used for statistical analysis.

### KIR ligand genotyping

DNA was isolated from 100 μl of peripheral blood using spin-column-based purification implemented with the DNeasy Blood and Tissue kit (Qiagen), and concentrations were measured using a Nanodrop 2000c spectrophotometer (Thermo Fisher). KIR ligand genotyping was performed using the Olerup SSP KIR HLA ligand typing kit (CareDx) following the manufacturer’s instructions. Reactions were analyzed on a 2 % (wt/vol) agarose gel prestained with GelRed (Biotium) in 0.5× TBE buffer. Gels were documented on a UV transilluminator and interpreted using the manufacturer-supplied reference tables. All lanes displayed the required control bands.

### Cell preparation for scATAC-seq

NK cells were enriched from peripheral blood mononuclear cells isolated from freshly drawn peripheral blood by magnetic depletion using micro-beads against CD3, CD14 and CD19. The enriched fraction was stained with the following fluorochrome-conjugated antibodies: CD7 BV786 (1:25; BD Biosciences), CD3 APC-eFluor 780 (1:50), CD14 APC-eFluor 780 (1:50) and CD19 APC-eFluor 780 (1:50; all Thermo Fisher), CD159a (NKG2A) biotin (1:50) and CD159c (NKG2C) PE (1:100; both REAfinity, Miltenyi Biotec). Fc receptors were blocked using Human TruStain FcX (1:50; BioLegend) for 15 min at 4 °C. Dead cells were excluded using Fixable Viability Dye eFluor 780 (Thermo Fisher). In a second step, cells were stained with combinations of the following nucleotide barcode-labeled antibodies (BioLegend) for 30 min at 4 °C: TotalSeq-A0084 CD56 (1:200), A0083 CD16 (1:500), A0436 anti-biotin (1:100), A0147 CD62L (1:100), A0168 CD57 (1:100), A0367 CD2 (1:1,000), A0801 CD337 (NKp30; 1:100), A0149 CD161 (1:100), A0420 CD158 (1:100), A0592 CD158b (1:100), A0599 CD158e1 (1:100), A0390 CD127 (1:400), A0902 CD328 (Siglec-7; 1:1,000), A0867 CD94 (1:100), A0896 CD85j (ILT2; 1:100), A0911 anti-phycoerythrin (1:100), A0250 KLRG1 (1:100), A0061 CD117 (1:100), A1018 HLA-DR, HLA-DP and HLA-DQ (1:400), A0355 CD137 (1:100), A0152 CD223 (1:100), A0366 CD184 (1:100), A0251 hashtag 1, A0252 hashtag 2, A0253 hashtag 3, A0254 hashtag 4, A0255 hashtag 5, A0256 hashtag 6, A0257 hashtag 7, A0258 hashtag 8, A0259 hashtag 9, A0260 hashtag 10, A0262 hashtag 12, A0263 hashtag 13, A0264 hashtag 14 and A0265 hashtag 15 (all 1:200–1:400). As described in the ASAP-seq and mitochondrial scATAC-seq (mtscATAC-seq) protocols, cells were fixed with 1% paraformaldehyde for 10 min. Fixation was quenched by adding glycin to a final concentration of 0.125 M, and cells were washed twice with PBS/bovine serum albumin (BSA). NK cells were sorted on a FACSAria II (BD Biosciences) as viable, single cells being CD3^−^CD14^−^CD19^−^CD7^+^, separating NKG2C^+^ and NKG2C^−^ cells. We used CD7 to sort for NK cells, as it is widely expressed on all NK cell subsets^58^ and enabled us to stain markers informative for NK cell differentiation, such as CD56 and CD16, with nucleotide barcode-labeled antibodies. After sorting, NKG2C^+^/ NKG2C^−^ NK cells were pooled and lysed for 3 min on ice using modified lysis buffer (10 mM Tris-HCl (pH 7.5), 10 mM NaCl, 3 mM MgCl_2_, 0.1% NP40 and 1% BSA). Afterward, lysed cells were washed with washing buffer (10 mM Tris-HCl (pH 7.5), 10 mM NaCl, 3 mM MgCl and 1% BSA) and resuspended in diluted nuclei buffer (10x Genomics). Lysis was confirmed by trypan blue staining, and cells were counted and further processed as described below.

### scATAC-seq

scATAC-seq libraries were prepared using the Chromium Next GEM single-cell ATAC reagent kit v1.1 following the manufacturer’s instructions with the modifications described in the ASAP-seq and mtscATAC-seq protocols to retrieve TotalSeq antibody-derived tags (ADTs), hashtag oligonucleotides (HTOs) and mtDNA^16–18^. The saved eluate from the silane bead elution and the supernatant of the first SPRIselect purification were combined for amplification of ADT/HTO libraries using the KAPA HiFi ready mix (Roche) with sample-specific index primers (Illumina small RNA RPIx/Truseq D7xx) and purified using SPRIselect reagent. Library size and quality were analyzed using a Fragment Analyzer (Advanced Analytical) before fragmentation and after final purification. Final library concentrations were measured on a Qubit 2.0 fluorometer (Thermo Fisher). Libraries were sequenced on a NextSeq500 or NovaSeq6000 sequencer (Illumina) using longer read1/2 configurations than suggested by 10x Genomics to improve mitochondrial genotyping (NextSeq500: R1 72 cycles, R2 72 cycles, I1 8 cycles, I2 16 cycles; NovaSeq6000: R1 88 cycles, R2 88 cycles, I1 8 cycles, I2 16 cycles).

### Cell preparation for scRNA-seq

NK cells were enriched, stained and sorted as described above for scATAC-seq (without fixation). NKG2C^+^ and NKG2C^−^ cells were separately labeled with different nucleotide-labeled hashtag antibodies (BioLegend). NKG2C^+^/NKG2C^−^ populations were pooled at a 1:1 ratio, and the cell concentration was adjusted to 1,000 cells per μl for further processing.

### scRNA-seq

scRNA-seq libraries were prepared using the Chromium Single Cell 3’ Reagent kit v2/v3.1 chemistry (10x Genomics) following the manufacturer’s instructions with the modifications required for recovery of ADTs and HTOs^19^. ADT and HTO libraries were amplified using the KAPA HiFi ready mix (Roche) with sample-specific index primers (Illumina small RNA RPIx/Truseq D7xx)^19^ and purified using SPRIselect reagent. Library size and quality were analyzed using a Fragment Analyzer (Advanced Analytical) before fragmentation and after final purification. Final library concentrations were measured on a Qubit 2.0 fluorometer (Thermo Fisher). Libraries were sequenced on a NextSeq500 or NovaSeq6000 sequencer (Illumina) using the read configurations recommended by 10x Genomics (v2: R1 26 cycles, R2 98 cycles, I1 8 cycles; v3: R1 28 cycles, R2 91 cycles, I1 8 cycles).

### Stimulation of NK cells from HCMV^–^ donors

RMA-S/HLA-E target cells were prepared by seeding cells in 24- to 96-well plates at a cell concentration of 2 × 10^6^ cells per ml in OptiMEM (Thermo Fisher) and irradiated with 3,000 cGy. Afterward, cells were pulsed either with the negative control peptide VMAPQSLLL or the activating peptide LFL (Peptides&Elephants) at a concentration of 300 μM for 6–12h.

For scATAC-seq, NK cells were isolated from freshly drawn blood by magnetic depletion of CD3^+^ cells, followed by enrichment of CD56^+^ cells. For read-out by flow cytometry, cells were isolated from buffy coats, and CD56^+^ cells were enriched using CD56 microbeads and were cryopreserved in FBS containing 10% DMSO. Viable CD56^+^CD3^−^ cells were sorted from the cryopreserved CD56-enriched fractions. Purified NK cells were cocultured for 12 h with peptide-pulsed RMA-S/HLA-E cells in complete medium supplemented with 10 ng ml^−1^ IL-15 (Miltenyi Biotec) and the respective peptides at 300 μM in the presence or absence of 10 ng ml^−1^ IL-12 (Miltenyi Biotec) and 100 ng ml^−1^ IL-18 (MBL International). Cells were stained with fluorochrome-labeled antibodies and either analyzed by flow cytometry or additionally stained with nucleotide barcode-labeled antibodies, including hashtag antibodies, marking experimental conditions and donors as described above before sorting for viable CD7^+^NKG2C^+^ cells. Sorted cells were pooled at equal numbers per condition and processed for scATAC-seq as described above.

### Removal of sensitive genetic variant information

Raw BAM files for all experiments were processed with BAMboozle^59^ to remove potentially identifying donor-related single nucleotide polymorphisms and indels by replacing them with the sequences present in the corresponding reference genomes. To enable reproducibility of the clonotype analysis based on mitochondrial mutations, the mitochondrial genotyping results were deposited with the raw and processed data for each experiment.

### Preprocessing of scATAC-seq data

Base call files were demultiplexed using cellranger-atac v1.2.0 mkfastq and bcl2fastq v2.20.0.422 into scATAC-seq and ADT/HTO libraries. scATAC-seq reads were mapped with cellranger-atac count to the GRCh38 reference genome, hardmasked for regions that would otherwise interfere with mapping to the mitochondrial genome^17^, generating count tables and fragment files as output for further analysis. ADT and HTO reads were preprocessed using ASAP to kite^18^, followed by pseudoalignment with kallisto^60^ to a mismatch map generated with kite and counting with bustools^61^.

### Integrated analysis of ex vivo scATAC-seq data

Peak sets called from different experiments were reduced to a joint peak set as a basis for combined analysis of individual experiments using Signac^62^. Cells were filtered for outliers based on nucleosome signal (<1–1.2), TSS enrichment (>2.5–4) and the frequency of reads in peaks (>55–70 %) and blacklisted regions (<0.0001). ADT and HTO counts were imported, centered log ratio normalized and joined with the scATAC-seq data. Normalized hashtag reads were used for exclusion of doublets and to demultiplex donors. Chromatin accessibility counts were normalized by term frequency inverse document frequency, data from different experiments were merged, and dimensionality was reduced by singular value decomposition. The resultant LSI was integrated across experiments using harmony^63^ and used as input into further dimensionality reduction by uniform manifold approximation (UMAP) and neighborhood graph-based clustering. A small population of non-NK cells, as judged by their lack of CD56 and CD16 expression concomitant with high levels of CD127 and CD117, was excluded. The merged and integrated dataset was used for peak calling using MACS2 (ref. ^64^) to enable more sensitive calling of cluster-specific open chromatin regions. This new assay was again processed by LSI, followed by anchor-based integration of donors^35^. The UMAP embeddings and clustering for final analysis were based on this integrated LSI. Unbiased clustering initially yielded three major clusters that were annotated based on surface protein expression, with early CD56^dim^ NK cells separated from CD56^bright^ NK cells by further subclustering. Gene activities per cell were calculated by summing up counts within the gene body and up to 2,000 bases upstream of the TSS.

For analysis of the global signatures, differential accessibility analysis of individual chromatin regions was performed using logistic regression with the number of peaks as latent variable^65^, while gene activities were assessed by Wilcoxon rank-sum test. For both, the log_2_ (fold change) threshold was set at 0.1, and significant hits from the direct comparison of CD56^bright^ to CD56^dim^ were added for visualization to include the CD56^dim^ signature that was mostly shared with adaptive NK cells. Averaged values were visualized in heat maps using tidyheatmap, an implementation of pheatmap.

To compare HCMV^+^ and HCMV^−^ donors, these were separated from the integrated dataset followed by UMAP embedding and clustering as described above. To directly compare the number of DARs between NKG2C^+^ and NKG2C^−^ cells, these populations were defined based on anti-PE ADT counts marking NKG2C surface expression and then downsampled to equal numbers (4,300 NKG2C^+^/NKG2C^−^ cells) for both donor groups. Further, NKG2C^+^ NK cells from HCMV^+^ donors were extracted, followed by UMAP embedding, and their cluster identities were assessed based on the signatures from the integrated analysis of the full dataset.

### Motif analysis

Position frequency matrices for TF motif analysis were downloaded from the JASPAR2020 human core database and amended by Stat-TF motifs from the murine database^66^. Motif activities per cell were calculated using chromVAR^27^. Enrichment of motifs in differentially accessible peaks between clusters was assessed using the hypergeometric test implemented in Signac. De novo motif analysis on the adaptive NK-cell-specific peaks was performed with homer v4.11 findMotifsGenome.pl using the standard parameters^67^.

### Mitochondrial genotyping and clonotypes

Mitochondrial genotyping was performed with mgatk^17^ in tenx mode using the barcodes identified as cells by cellranger. Only cells with at least 5× coverage of the mitochondrial genome were included in the analysis, achieving a median coverage of 11–20× across experiments. As sensitivity and positive predictive value are relatively independent of coverage for mutations with high heteroplasmy^17^, those could be confidently detected at this coverage. Using a combination of highly abundant, homoplasmic mitochondrial mutations characteristic of each donor, we noted that donor demultiplexing was slightly incomplete in some cases, so we used these mutations as additional donor barcodes to further improve demultiplexing before the donor-specific analysis. Mitochondrial mutations called by mgatk were filtered for high-confident variants detected in at least three cells, with strand concordance of >65% and a variance-to-mean ratio of >0.01. Clonotypes were identified by clustering on a neighborhood graph constructed on mitochondrial mutation frequency using the Euclidean distance metric, because we noted that the majority of adaptive clonotypes were defined by mutations with high allele frequencies and thereby achieved better clonotype resolution than with the cosine distance metric originally suggested for mtscATAC-seq^17^. Clustering parameters were empirically optimized per donor so that clonotypes were defined by approximately one mutation, and only those clonotypes were included that had at least one significantly enriched mutation. Clonotypes only defined by differential frequency of highly abundant mutations were excluded from the analysis. Association of clonotypes to clusters defined by their chromatin accessibility profiles was analyzed by χ^2^ test and comparing the resultant false discovery rates (FDRs) to a randomly permuted matrix^17^. Analysis of clonotype association to open chromatin regions was performed using a χ^2^ test on the binarized accessibility matrix and compared to a randomly permuted matrix.

### Donor-specific and donor-unique signatures

Individual HCMV^+^ donors were analyzed based on the peak set called for the integrated dataset. Both time points were included in this analysis and integrated using harmony^62^. Dimensionality reduction and clustering was performed for each individual donor as described above. To analyze overlaps and differences between the signatures defining clusters across donors, differential accessibility analysis was performed per donor as described for the integrated dataset, and overlaps were visualized using eulerr. To assess the actual signatures, adaptive subclusters defined in each donor were merged, and averaged accessibility per cluster was visualized with tidyheatmap. Differential gene scores and motif activities between the two emerging adaptive subcluster groups were analyzed by directly comparing them to each other within the merged object. To assess similarity of their signatures to those of CD56^bright^/early CD56^dim^ and CD56^dim^ NK cells, fold changes of the adaptive subcluster group-defining DARs were plotted for both comparisons and analyzed for linear correlation.

### Cluster heterogeneity

Cluster heterogeneity was assessed based on the median Euclidian distance of 200 randomly sampled cells per cluster to their 10 nearest neighbors in LSI space (components 2–30). Sampling was repeated 100 times, and results were plotted as box plots of the distribution of median distances of all simulations. The *k* parameter was varied from 5 to 30 to test for robustness.

### Longitudinal analysis

The two time points for donors P2, P3 and P4 were separated from the merged, harmony-integrated objects, and the signatures defining adaptive subclusters were analyzed separately for each time point using hierarchical clustering and visualization with tidyheatmap. Allele frequency of clonotype-defining mutations was visualized for each time point. To analyze the stability of clonotypes over time, only clonotypes that had an association with chromatin accessibility-defined clusters with an FDR of <0.05 were included; these were further curated by assessment of their association with adaptive subclusters in UMAP embeddings. Stability of clonotypes significantly associated with adaptive subclusters was analyzed by testing association of clonotypes with time points by Fisher’s exact test. The stability of the total clonotype distribution was further assessed by calculating their log_2_ (fold change) values between time points and comparing the distribution to a randomly permuted clonotype–time point relationship by Kolmogorov–Smirnov test.

### Analysis of scATAC-seq data after activation

Quality control, joining of ADT, HTO and scATAC-seq counts, demultiplexing, peak calling on the full dataset with MACS2, normalization and dimensionality reduction by LSI were performed as described for the ex vivo analysis. The two analyzed donors were integrated with harmony, and the corrected LSI was used for UMAP embedding and clustering. After inspection of the distribution of cells from the different conditions and donors, the majority of cells were clearly separated based on whether they had received peptide or cytokine stimulation, but we also noted two smaller clusters, each enriched for cells from one donor, respectively, in which cells from different conditions were mixed, likely driven by underlying donor-specific signatures that were not corrected by harmony integration. To analyze stimulus-induced changes in chromatin accessibility, we focused the analysis on the majority of cells that clustered by conditions and irrespective of donor origin. The cell barcodes used for the final analysis were deposited together with the raw and processed data. After clustering these cells at higher resolution, we annotated clusters according to a clear enrichment of cells derived from different conditions (Extended Data Fig. 4b). To assess the similarity of the in vitro induced signatures to those defining NK cell subsets ex vivo, the MACS2-called peak set of the ex vivo analysis was quantified for the cells stimulated in vitro, and the two datasets were integrated using anchor-based integration, setting the ex vivo dataset as reference and the in vitro dataset as query^35^. Integration anchors were then used to transfer cluster labels from the ex vivo dataset to the in vitro dataset, and the per-cell prediction scores were plotted for each cluster. To identify which individual chromatin regions overlap between adaptive NK cells ex vivo and the cells stimulated with cytokines and peptide, fold changes for DARs overlapping between the comparisons of CD56^dim^ cells to adaptive NK cells ex vivo and control cells to LFL + IL-12+IL-18 cells in vitro were analyzed for linear correlation.

### Preprocessing of scRNA-seq data

Base call files were demultiplexed using cellranger v3.0.2 mkfastq and bcl2fastq v2.20.0.422 into scRNA-seq and ADT/HTO libraries. scRNA-seq reads were mapped to the hg19 reference genome with cellranger count, generating gene-per-cell count tables as output for further analysis. ADT and HTO reads were either quantified with CITE-Seq-Count^68^ or pseudoaligned with kallisto^60^ to a mismatch map generated with kite and counted with bustools^61^.

### Downstream analysis of scRNA-seq data

scRNA-seq counts were imported into Seurat^69^ and filtered for outliers regarding frequency of mitochondrial transcripts (v2: <5–6%; v3: <10%), total number of transcripts (v2: <6,000; v3: <10,000) and number of genes (v2: >500–800, <2,000–2,500; v3: >1,000, <5,000) per cell. Contaminating erythrocytes and B cells were excluded based on the expression of *HBA1/HBA2* and *IGJ*, respectively. ADT/HTO counts were imported, centered log ratio normalized and joined with the scRNA-seq data. Normalized hashtag reads were used for exclusion of doublets and to demultiplex NKG2C^+^/NKG2C^−^ populations and donors. Initially, donors were analyzed individually. Counts were normalized with scran^70^ and used for principal-component analysis (PCA) on the 2,000 most variable genes. The number of principal components used for UMAP embedding and clustering was chosen based on elbow plots for each individual. After initial high-resolution clustering, a small population of non-NK cells, as judged by their lack of CD56 expression concomitant with high levels of *IL7R, GATA3, IL2RA* and *CD40LG*, were excluded. The PCA was recalculated on the remaining cells and used as input for UMAP embedding and clustering as before. HCMV^+^ and HCMV^−^ donors were then integrated separately based on the variable features overlapping between HCMV^+^ donors by using anchor-based integration. The integrated data were scaled, regressing out the number of transcripts per cell. The HCMV^+^/HCMV^–^ objects were merged, and the integrated data were rescaled, also regressing out the number of transcripts per cell. A PCA was performed on the shared variable features and was used as input for UMAP embedding and clustering. The normalized, non-integrated RNA assay was used for all differential expression analyses. For differential expression analysis between NKG2C^+^ and NKG2C^−^ populations in HCMV^+^ and HCMV^−^ donors, cells were downsampled to equal numbers per population and donor group (6,000 cells each), and only genes that were detected with a minimum count of 100 in both objects were included. Further, NKG2C^+^ NK cells from HCMV^+^ donors were extracted using hashtag counts, followed by UMAP embedding. Signatures from the integrated analysis were assessed to confirm the cluster identities.

### Integration of scRNA-seq and scATAC-seq data

Fully processed and donor-integrated datasets of both modalities were integrated by anchor-based integration as implemented in Seurat^35,69^ using the integrated RNA assay as reference and the gene scores calculated from scATAC-seq data as query. Integration anchors were used to impute RNA expression of clusters defined by chromatin accessibility and inspected for consistency of key marker genes. Imputed RNA expression was assessed for correlation with chromatin accessibility to identify links between individual chromatin regions and gene expression.

### Statistical analysis and reproducibility

Reported *P* values were corrected for multiple testing. No statistical methods were used to predetermine sample sizes, but our sample sizes are similar to those reported in previous publications^20,22^. Data distribution was assumed to be normal, but this was not formally tested. No randomization was performed. Data collection and analysis were not performed blind to the conditions of the experiments. No data points were excluded.

### Reporting summary

Further information on research design is available in the Nature Research Reporting Summary linked to this article.

## Extended Data

## Supplementary Material

The online version contains supplementary material available at https://doi.org/10.1038/s41590-022-01327-7.

Supplementary Material

## Data Availability

scRNA-seq and scATAC-seq BAM files and processed data as fragment files, gene count table and antibody count tables have been deposited in the Gene Expression Omnibus under accession code GSE197037. Single-nucleotide variants and indels were removed from BAM files as described in the Methods. To enable reproducibility of the clonotype analysis based on mitochondrial mutations, the mgatk results were deposited for each experiment. scATAC-seq data were mapped to the GRCh38 (GCF_000001405.39) reference genome, and scRNA-seq data were mapped to the hg19 (GCF_000001405.25) reference genome, as supplied by 10x Genomics. Source data are provided with this paper.

## Code availability

All code has been deposited in GitHub (https://github.com/timorueckert/Clonal_NK).
