# Supplementary material for: Clonal expansion and epigenetic inheritance of long-lasting NK cell memory
Source: Nat Immunol. Author manuscript; Available in PMC 2022 Nov 25. (PMC9663309; doi:10.1038/s41590-022-01327-7)
Supplement: Supplementary Material [file EMS156160-supplement-Supplementary_Material.pdf]

nature immunology

Article

https://doi.org/10.1038/s41590-022-01327-7

Clonal expansion and epigenetic inheritance of long-lasting NK cell memory

In the format provided by the authors and unedited

Supplementary Table 1 Healthy blood donor characteristics. HCMV serostatus was determined by anti-HCMV IgG ELISA (see Methods).

Donor ID	HCMV Serostatus	Sex	Age at inclusion	scATAC	scRNA
P1	+	m	45	x	x
P2	+	m	27	x	x
P3	+	w	29	x	x
P4	+	w	27	x	x
P5	+	m	28		x
N1	-	w	30	x	x
N2	-	m	29	x	x
N3	-	m	33	x	
N4	-	w	29	x	

Supplementary Table 2 KIR ligand genotyping results. KIR ligand genotypes were determined by sequence-specific priming PCR (see Methods).

Donor ID	HLA-A	HLA-B	HLA-C	Educating KIRs
P1	$Bw4^{-}$	$Bw4^{+}$Ile80	C1/C2	KIR2DL1, KIR2DL2/3, KIR3DL1
P2	$Bw4^{+}$	$Bw4^{+}$Ile80	C2/C2	KIR2DL1, KIR3DL1
P3	$Bw4^{+}$	$Bw4^{+}$Thr80	C1/C2	KIR2DL1, KIR2DL2/3, KIR3DL1
P4	$Bw4^{-}$	$Bw4^{-}$	C1/C2	KIR2DL1, KIR2DL2/3
