# Supplementary material for: Clonal expansion and epigenetic inheritance of long-lasting NK cell memory
Source: Nat Immunol. 2022 Oct 26;23(11):1551–63. doi: 10.1038/s41590-022-01327-7 (PMC9663309; doi:10.1038/s41590-022-01327-7)
Supplement: Supplementary file 1 [file 41590_2022_1327_MOESM2_ESM.pdf]

nature portfolio

Corresponding author(s): Chiara Romagnani, Timo Rückert

Last updated by author(s): Aug 27, 2022

Reporting Summary

Nature Portfolio wishes to improve the reproducibility of the work that we publish. This form provides structure for consistency and transparency in reporting. For further information on Nature Portfolio policies, see our Editorial Policies and the Editorial Policy Checklist.

Statistics

For all statistical analyses, confirm that the following items are present in the figure legend, table legend, main text, or Methods section.

n/a	Confirmed	
☐	☒	The exact sample size (*n*) for each experimental group/condition, given as a discrete number and unit of measurement
☐	☒	A statement on whether measurements were taken from distinct samples or whether the same sample was measured repeatedly
☐	☒	The statistical test(s) used AND whether they are one- or two-sided<br>*Only common tests should be described solely by name; describe more complex techniques in the Methods section.*
☒	☐	A description of all covariates tested
☐	☒	A description of any assumptions or corrections, such as tests of normality and adjustment for multiple comparisons
☐	☒	A full description of the statistical parameters including central tendency (e.g. means) or other basic estimates (e.g. regression coefficient) AND variation (e.g. standard deviation) or associated estimates of uncertainty (e.g. confidence intervals)
☐	☒	For null hypothesis testing, the test statistic (e.g. *F*, *t*, *r*) with confidence intervals, effect sizes, degrees of freedom and *P* value noted<br>*Give P values as exact values whenever suitable.*
☒	☐	For Bayesian analysis, information on the choice of priors and Markov chain Monte Carlo settings
☒	☐	For hierarchical and complex designs, identification of the appropriate level for tests and full reporting of outcomes
☐	☒	Estimates of effect sizes (e.g. Cohen's *d*, Pearson's *r*), indicating how they were calculated

Our web collection on statistics for biologists contains articles on many of the points above.

Software and code

Policy information about availability of computer code

Data collection	For in vitro cultures NK cells were sorted using a FACS Aria II (BD Biosciences). Flow cytometry data was acquired using LSR Fortessa (BD Biosciences). Sequencing libraries were sequenced on a NextSeq500 or NovaSeq6000 sequencer (Illumina).
Data analysis	Flow cytometry data was analyzed using FlowJo v 10.7.1. Statistical analysis was performed GraphPad Prism v8.4.3. Analysis of sequencing data was performed as detailed in the methods section, using the following software packages:<br>Python 3.7.9<br>cellranger-atac v1.2.0<br>cellranger v3.0.2<br>ASAP to kite v2<br>kallisto v0.46.0<br>bustools v0.39.2<br>bamboozle v0.5.0<br>CITE-seq count v1.4.1<br>mgatk v0.5.9<br>R v4.1.0<br>Seurat v4.0.6<br>Signac v1.3.0<br>Harmony v0.1.0<br>chromVAR 1.14.0<br>tidyheatmap v0.0.0.9000<br>pheatmap v1.0.12<br>eulerr v6.1.0<br>Homer v4.11

Analysis code was deposited at GitHub: https://github.com/timorueckert/Clonal_NK

For manuscripts utilizing custom algorithms or software that are central to the research but not yet described in published literature, software must be made available to editors and reviewers. We strongly encourage code deposition in a community repository (e.g. GitHub). See the Nature Portfolio guidelines for submitting code & software for further information.

Data

Policy information about availability of data

All manuscripts must include a data availability statement. This statement should provide the following information, where applicable:

- Accession codes, unique identifiers, or web links for publicly available datasets
- A description of any restrictions on data availability
- For clinical datasets or third party data, please ensure that the statement adheres to our policy

scRNA and scATAC-seq bam-files, as well as processed data as fragment files, gene count table and antibody count tables have been deposited at GEO (accession number GSE197037) and are publicly available as of the date of publication. Single-nucleotide variants and indels were removed from bam files as described in the detailed methods section. To enable reproducibility of the clonotype analysis based on mitochondrial mutations, the mgatk results were deposited for each experiment. scATAC-seq data were mapped to the GRCh38 (GCF_000001405.39), scRNA-seq data to hg19 (GCF_000001405.25) as supplied by 10x genomics.

Field-specific reporting

Please select the one below that is the best fit for your research. If you are not sure, read the appropriate sections before making your selection.

☒ Life sciences ☐ Behavioural & social sciences ☐ Ecological, evolutionary & environmental sciences

For a reference copy of the document with all sections, see nature.com/documents/nr-reporting-summary-flat.pdf

Life sciences study design

All studies must disclose on these points even when the disclosure is negative.

Sample size	No sample size calculation was performed but our sample sizes are similar to those reported in previous publications. For the scATAC- and scRNA-seq, we decided to include more HCMV+ donors as the focus of the study was on adaptive NK cells that were specifically present in HCMV+ donors whereas conventional NK cells were well represented in both HCMV+ and HCMV- donors.
Data exclusions	No data was excluded.
Replication	scATAC-seq ex vivo data were collected in 6 independent experiments. scRNA-seq ex vivo data were collected in four independent experiments. In vitro stimulation followed by scATAC-seq is from one experiment with two indepedently stimulated donors, read out by flow cytometry was performed in three independent experiments with three donors each, all of which successfully replicated the results.
Randomization	Donors were allocated into groups based on HCMV-serostatus. No additional group allocation or randomization was performed.
Blinding	Investigators were not blinded to group allocation. The same unbiased analysis strategy was applied for all comparative analysis of the HCMV+ and HCMV- groups, including equal enrichment of NKG2C+ and NKG2C- cells.

Reporting for specific materials, systems and methods

We require information from authors about some types of materials, experimental systems and methods used in many studies. Here, indicate whether each material, system or method listed is relevant to your study. If you are not sure if a list item applies to your research, read the appropriate section before selecting a response.

Materials & experimental systems

n/a	Involved in the study	
☐	☒	Antibodies
☐	☒	Eukaryotic cell lines
☒	☐	Palaeontology and archaeology
☒	☐	Animals and other organisms
☐	☒	Human research participants
☒	☐	Clinical data
☒	☐	Dual use research of concern

Methods

n/a	Involved in the study	
☒	☐	ChIP-seq
☐	☒	Flow cytometry
☒	☐	MRI-based neuroimaging

Antibodies

Antibodies used	TotalSeq™-A0084 anti-human CD56 (NCAM) Biolegend AB_2734445 (BioLegend Cat#392421) 1:200

Antibodies used

TotalSeq™-A0083 anti-human CD16 Biolegend AB_2734255 (BioLegend Cat#302061) 1:500
TotalSeq™-A0436 anti-Biotin Biolegend AB_2801086 (BioLegend Cat#409008) 1:100
TotalSeq™-A0147 anti-human CD62L Biolegend AB_2750365 (BioLegend Cat#304847) 1:100
TotalSeq™-A0168 anti-human CD57 Biolegend AB_2810588 (BioLegend Cat#393319) 1:100
TotalSeq™-A0367 anti-human CD2 Biolegend AB_2783172 (BioLegend Cat#309229) 1:1000
TotalSeq™-A0801 anti-human CD337 (NKp30) Biolegend AB_2800852 (BioLegend Cat#325221) 1:100
TotalSeq™-A0149 anti-human CD161 Biolegend AB_2749998 (BioLegend Cat#339945) 1:100
TotalSeq™-A0420 anti-human CD158 (KIR2DL1/S1/S3/S5) Biolegend AB_2800901 (BioLegend Cat#339515) 1:100
TotalSeq™-A0592 anti-human CD158b (KIR2DL2/L3, NKAT2) Biolegend AB_2800818 (BioLegend Cat#312615) 1:100
TotalSeq™-A0599 anti-human CD158e1 (KIR3DL1, NKB1) Biolegend AB_2800819 (BioLegend Cat#312723) 1:100
TotalSeq™-A0390 anti-human CD127 (IL-7Rα) Biolegend AB_2734366 (BioLegend Cat#351352) 1:400
TotalSeq™-A0902 anti-human CD328 (Siglec-7) Biolegend AB_2832662 (BioLegend Cat#339217) 1:1000
TotalSeq™-A0867 anti-human CD94 Biolegend AB_2814142 (BioLegend Cat#305521) 1:100
TotalSeq™-A0896 anti-human CD85j (ILT2) Biolegend AB_2814225 (BioLegend Cat#333723) 1:100
TotalSeq™-A0911 anti-phycoerythrin (PE) Biolegend AB_2820078 (BioLegend Cat#408109) 1:100
TotalSeq™-A0250 anti-mouse/human KLRG1 (MAFA) Biolegend AB_2800648 (BioLegend Cat#138431) 1:100
TotalSeq™-A0061 anti-human CD117 (c-kit) Biolegend AB_2734287 (BioLegend Cat#313241) 1:100
TotalSeq™-A1018 anti-human HLA-DR, DP, DQ Biolegend AB_2832712 (BioLegend Cat#361717) 1:400
TotalSeq™-A0355 anti-human CD137 (4-1BB) Biolegend AB_2783173 (BioLegend Cat#309835) 1:100
TotalSeq™-A0152 anti-human CD223 (LAG-3) Biolegend AB_2749999 (BioLegend Cat#369333) 1:100
TotalSeq™-A0366 anti-human CD184 (CXCR4) Biolegend AB_2800790 (BioLegend Cat#306531) 1:100
TotalSeq™-A0251 anti-human Hashtag 1 Biolegend AB_2750015 (BioLegend Cat#394601) 1:200-1:400
TotalSeq™-A0252 anti-human Hashtag 2 Biolegend AB_2750016 (BioLegend Cat#394603) 1:200-1:400
TotalSeq™-A0253 anti-human Hashtag 3 Biolegend AB_2750017 (BioLegend Cat#394605) 1:200-1:400
TotalSeq™-A0254 anti-human Hashtag 4 Biolegend AB_2750018 (BioLegend Cat#394607) 1:200-1:400
TotalSeq™-A0255 anti-human Hashtag 5 Biolegend AB_2750019 (BioLegend Cat#394609) 1:200-1:400
TotalSeq™-A0256 anti-human Hashtag 6 Biolegend AB_2750020 (BioLegend Cat#394611) 1:200-1:400
TotalSeq™-A0257 anti-human Hashtag 7 Biolegend AB_2750021 (BioLegend Cat#394613) 1:200-1:400
TotalSeq™-A0258 anti-human Hashtag 8 Biolegend AB_2750022 (BioLegend Cat#394615) 1:200-1:400
TotalSeq™-A0259 anti-human Hashtag 9 Biolegend AB_2750023 (BioLegend Cat#394617) 1:200-1:400
TotalSeq™-A0260 anti-human Hashtag 10 Biolegend AB_2750024 (BioLegend Cat#394619) 1:200-1:400
TotalSeq™-A0262 anti-human Hashtag 12 Biolegend AB_2750025 (BioLegend Cat#394623) 1:200-1:400
TotalSeq™-A0263 anti-human Hashtag 13 Biolegend AB_2750026 (BioLegend Cat#394625) 1:200-1:400
TotalSeq™-A0264 anti-human Hashtag 14 Biolegend AB_2750027 (BioLegend Cat#394627) 1:200-1:400
TotalSeq™-A0265 anti-human Hashtag 15 Biolegend AB_2750028 (BioLegend Cat#394629) 1:200-1:400
PE/Dazzle™ 594 anti-human CD56 Biolegend AB_2563564 (BioLegend Cat#318348) 1:200
PE/Cyanine7 anti-human CD137 (4-1BB) Biolegend AB_2207741 (BioLegend Cat#309818)
Brilliant Violet 605™ anti-human CD57 Recombinant Antibody AB_2728426 (BioLegend Cat. No. 393304) 1:25
BV786 Mouse Anti-Human CD7 BD Biosciences AB_2740589 (BD Biosciences Cat#740964) 1:25
CD159a (NKG2A) Antibody, anti-human, Biotin, REAfinity Miltenyi Biotec AB_2783969 (Miltenyi Biotec Cat#130-114-090) 1:50
CD159c (NKG2C) Antibody, anti-human, PE, REAfinity™ Miltenyi Biotec AB_2751866 (Miltenyi Biotec Cat#130-119-814) 1:100
CD159a (NKG2A) Antibody, anti-human, PE-Vio770, REAfinity Miltenyi Biotec AB_2655388 (Miltenyi Biotec Cat#130-105-647) 1:50
CD3 Monoclonal Antibody (SK7), APC-eFluor 780 Invitrogen AB_10717514 (ThermoFisher Cat#47-0036-42) 1:50
CD14 Monoclonal Antibody (61D3), APC-eFluor 780 Invitrogen AB_1834358 (Cat#47-0149-42) 1:50
CD19 Monoclonal Antibody (HIB19), APC-eFluor 780, Invitrogen AB_1582230 (Cat#47-0199-42) 1:50
Anti-FcεRI Antibody, γ subunit-FITC Merck Cat#FCABS400F 1:50
CD337 (NKp30) Monoclonal Antibody (AF29-4D12), eFluor 450 ThermoFisher AB_2574058 (Cat#48-3379-42) 1:25
BUV737 Mouse Anti-Human CD56 (NCAM-1) BD Biosciences AB_2871176 (BD Biosciences Cat#741842) 1:50
BUV805 Mouse Anti-Human CD3 BD Biosciences (BD Biosciences Cat#612896) 1:50
BUV496 Mouse Anti-Human CD16 BD Biosciences AB_2870224 (BD Biosciences Cat#612944) 1:50
BV421 Mouse Anti-Human CD337 (NKp30) BD Biosciences AB_2738171 (BD Biosciences Cat#563385) 1:25
BUV395 Streptavidin BD Biosciences AB_2869553 (BD Biosciences Cat#564176) 1:100

Validation

All purchased antibodies were validated by their manufacturers and further in-house testing.
- Miltenyi Biotec, https://www.miltenyibiotec.com/DE-en/lp/antibody-validation-improved-reproducibility.html
Three pillars of antibody validation: 1. Antibody reproducibility and consistency (Pure antibody products & Lot-to-lot consistent performance); 2. Antibody specificity (Epitope competition assay,Knockout validation via targeted genome editing & RNAi knockdown); 3. Antibody sensitivity (Functional testing of every product prior to release, Performance comparison & Compatibility with fixation).

- BioLegend, https://www.biolegend.com/en-us/quality/quality-control
Flow Cytometry Reagents
Specificity testing of 1-3 target cell types with either single- or multi-color analysis (including positive and negative cell types).
Once specificity is confirmed, each new lot must perform with similar intensity to the in-date reference lot. Brightness (MFI) is evaluated from both positive and negative populations.
Each lot product is validated by QC testing with a series of titration dilutions.
TotalSeq™ Antibodies
Bulk lots are tested by PCR and sequencing to confirm the oligonucleotide barcodes. They are also tested by flow cytometry to ensure the antibodies recognize the proper cell populations.
Bottled lots are tested by PCR and sequencing to confirm the oligonucleotide barcodes.

- BD Biosciences, https://www.biocompare.com/Antibody-Manufacturing/355107-Antibody-Manufacturing-Perspectives-BDBioscience/
We conduct quality control (QC) testing in primary model systems to ensure biological accuracy in an ISO 9001 certified facility. BD carefully selects and characterizes antibody content in product development and tests in relevant primary model systems to ensure

biological accuracy. BD conducts rigorous QC testing of each antibody lot tested side-by-side with a previously produced lot as reference. Our product development process includes testing on a combination of primary cells, cell lines and/or transfectant cell models with relevant controls using multiple immunoassays to ensure biological accuracy. We also perform multiplexing with additional antibodies to interrogate antibody staining in multiple cell populations. BD believes antibody validation is critical to ensure accurate scientific results. Both the consumer and the reagent provider share the responsibility for reproducible science.

- Invitrogen, https://www.thermofisher.com/de/de/home/life-science/antibodies/invitrogen-antibody-validation.html
To help ensure superior antibody results, we've expanded our specificity testing methodology using a 2-part approach for advanced verification. Part 1—Target specificity verification. This helps ensure the antibody will bind to the correct target. Our antibodies are being tested using at least 1 of the following methods to ensure proper functionality in researcher's experiments.
Part 1—Target specificity verification
This helps ensure the antibody will bind to the correct target. Our antibodies are being tested using at least one of the following methods to ensure proper functionality in researcher's experiments.

Knockout—expression testing using CRISPR-Cas9 cell models
Knockdown—expression testing using RNAi to knockdown gene of interest
Independent antibody verification (IAV)—measurement of target expression is performed using two differentially raised antibodies recognizing the same protein target
Cell treatment—detecting downstream events following cell treatment
Relative expression—using naturally occurring variable expression to confirm specificity
Neutralization—functional blocking of protein activity by antibody binding
Peptide array—using arrays to test reactivity against known protein modifications
SNAP-ChIP™—using SNAP-ChIP to test reactivity against known protein modifications
Immunoprecipitation-Mass Spectrometry (IP-MS)—testing using immunoprecipitation followed by mass spectrometry to identify antibody targets
Part 2—Functional application validation.
These tests help ensure the antibody works in a particular application(s) of interest, which may include
(but are not limited to):

Western blotting
Flow cytometry
ChIP
Immunofluorescence imaging
Immunohistochemistry

-Merck
Evaluated by flow cytometry using RBL cells.

Eukaryotic cell lines

Policy information about cell lines

Cell line source(s)	RMA-S/HLA-E were provided by J. Coligan, National Institutes of Health
Authentication	Expression of HLA-E was frequently validated by flow cytometry, no further authentication was performed.
Mycoplasma contamination	RMA-S/HLA-E were tested negative for mycoplasma contamination.
Commonly misidentified lines (See ICLAC register)	No commonly misidentified cell lines were used in the study.

Human research participants

Policy information about studies involving human research participants

Population characteristics	5 HCMV+ and 4 HCMV- healthy blood donors were included in the sequencing study, age and gender are listed in Supplementary Table. 1.
Recruitment	Healthy participants were initially randomly recruited for screening of HCMV serostatus and presence of NKG2C+ NK cell expansions. HCMV+ donors were selected based on the presence of NKG2C+ NK cell expansions as judged by flow cytometry, which might have biased the donor selection towards donors with macroscopically visible adaptive NK cell expansions.
Ethics oversight	The Charité ethics committee approved the study (EA4/196/18 and EA4/059/17).

Note that full information on the approval of the study protocol must also be provided in the manuscript.

Flow Cytometry

Plots

Confirm that:

☒ The axis labels state the marker and fluorochrome used (e.g. CD4-FITC).

☒ The axis scales are clearly visible. Include numbers along axes only for bottom left plot of group (a 'group' is an analysis of identical markers).

☒ All plots are contour plots with outliers or pseudocolor plots.

☒ A numerical value for number of cells or percentage (with statistics) is provided.

Methodology

Sample preparation	PBMCs were isolated from freshly drawn peripheral blood of healthy donors or from buffy coats obtained from DRK Blutspendedienst Nord-Ost, Dresden, Germany by density-gradient centrifugation (Ficoll Paque Plus, GE Healthcare) and either processed immediately or cryopreserved in FBS containing 10 % DMSO. Dead cells were excluded using Fixable Viability Dye eFluor780 (ThermoFisher), or Zombie Aqua Fixable Viability Kit (BioLegend). Cells were stained with fluorochrome conjugated antibodies for 15 min at room temperature, for sorting, cells were stained at 4 °C. Staining with nucleotide-labeled antibodies was performed for 30 min at 4 °C. For sequencing, NK cells were pre-enriched by magnetic depletion of cells expressing CD3, CD14 or CD19 (Miltenyi Biotec). For in vitro stimulation analyzed by flow cytometry, NK cells were pre-enriched with CD56 microbeads (Miltenyi Biotec).
Instrument	LSR Fortessa, FACSAria II (both BD Biosciences)
Software	FlowJo v 10.7.1
Cell population abundance	Purity of the sorted cell population was analyzed in a post-sort reanalysis and was consistently >90%. NKG2C+ and NKG2C- cells were equally enriched for HCMV+/- donor groups.
Gating strategy	For sequencing, NK cells were sorted as CD3- CD14- CD19- CD7+ NKG2C+/- cells. For in vitro stimulation and measurement by flow cytometry, NK cells were sorted as CD56+ CD3- from CD56-enriched fractions.

☒ Tick this box to confirm that a figure exemplifying the gating strategy is provided in the Supplementary Information.
